# mRNA-laden LNP-enabled in situ CAR-macrophage alleviates liver fibrosis via inhibiting activated HSCs and modulating the immune microenvironment

**DOI:** 10.1073/pnas.2534673123

**Published:** 2026-05-29

**Authors:** Xin Huang, Junfeng Hao, Shuo Wang, Botian Deng, Peng Wang, Qiuyu Zhao, Hongbo Liu, Jiahe Wang

**Affiliations:** ^a^Department of Family Medicine, Shengjing Hospital of China Medical University, Shenyang 110022, China; ^b^ Department of Nephrology, and Guangdong Provincial Key Laboratory of Autophagy and Major Chronic Non-communicable Diseases, Affiliated Hospital of Guangdong Medical University, Zhanjiang 524001, China; ^c^Department of Cardiology, Shengjing Hospital of China Medical University, Shenyang 110022, China; ^d^ Department of Key Laboratory of Ministry of Education for Traditional Chinese Medicine Viscera-State Theory and Applications, Liaoning University of Traditional Chinese Medicine, Shenyang 110847, China; ^e^Third Department of Respiratory, Shengjing Hospital of China Medical University, Shenyang 110022, China

**Keywords:** FAP-CAR, liver fibrosis, LNPs, ECM, macrophages

## Abstract

Liver fibrosis, marked by an abnormal buildup of extracellular matrix (ECM), poses a major health threat. Myofibroblasts, predominantly derived from hepatic stellate cells (HSCs) and portal fibroblasts, are the primary drivers of ECM synthesis. Fibroblast activation protein (FAP), highly expressed by activated HSCs, is a pivotal player in the pathogenesis of liver fibrosis. Delineating the mechanisms underlying HSC activation and devising strategies to curb their hyperactivity are paramount for the management and prevention of liver fibrosis. In this study, we explored a pioneering therapeutic approach leveraging CD163 antibody-conjugated liposomal nanoparticles (LNPs) encapsulating FAP-specific chimeric antigen receptor macrophage (CAR-M) mRNA (αCD163/LNP-FAPCAR). These LNPs are designed to selectively transduce liver macrophages, facilitating the in situ generation of FAP-specific CAR-modified macrophages (FAPCAR-M). Our findings revealed that these LNPs efficiently transduced macrophages, augmenting their phagocytic capabilities toward target cells. This resulted in a significant reduction of ECM and a concomitant enhancement of liver fibrosis resolution. The overarching goal is to precisely target and neutralize hyperactive fibroblasts, offering a promising avenue for treating liver fibrosis.

## Significance

Liver fibrosis is fundamentally driven by the activation of hepatic stellate cells (HSCs), wherein activated HSCs characterized by elevated fibroblast activation protein (FAP) expression serve as pivotal pathogenic effectors promoting excessive extracellular matrix (ECM) deposition and hepatic microenvironmental perturbation. Current approaches fall short in precisely targeting these pathogenic cells. Herein, we engineered a CD163-targeted lipid nanoparticle (LNP) platform capable of delivering FAP-chimeric antigen receptor (CAR) messenger RNA (mRNA) to hepatic macrophages, thereby programming in situ-generated chimeric antigen receptor macrophagecells for selective recognition and clearance of activated HSCs. This αCD163/LNP-FAPCAR system markedly attenuated fibrotic burden while restoring hepatic microenvironmental integrity, heralding a paradigm shift toward macrophage-based immunotherapy for chronic liver disease.

Liver fibrosis is a chronic, progressive pathological process characterized by excessive accumulation of extracellular matrix (ECM) in the liver. It is associated with high morbidity and mortality rates and is a major component of the global disease burden (1). Under homeostatic conditions, hepatocytes engage in a balanced synthesis and degradation of collagen proteins and ECM constituents, ensuring the stability and functionality of liver tissue (2). This equilibrium, however, can be perturbed by factors such as excessive alcohol intake, viral hepatitis (e.g., HBV or HCV), autoimmune disorders, and other etiologies that incite hepatic injury (3).

Myofibroblasts are the principal cellular entities responsible for the synthesis of collagen and, more broadly, the ECM in the liver and other organs. Predominantly within the hepatic environment, these cells differentiate primarily from hepatic stellate cells (HSCs) and portal fibroblasts (4). It is imperative to note that HSCs are critically implicated in the initiation and progression of liver fibrosis, underscoring their significant role in hepatic pathophysiology (3). Fibroblast activation protein (FAP) exhibits elevated expression in a spectrum of malignancies, including breast, lung, and pancreatic cancers, and is also upregulated in chronic hepatic pathologies, notably cirrhosis (5, 6). FAP is minimally present in healthy adult livers, but its expression increases significantly in the fibrotic liver’s active matrix (3). During liver fibrosis, FAP is predominantly expressed by activated HSCs. Studies have demonstrated that inhibiting FAP or genetically eliminating it can lessen liver fibrosis in animal models (7).

Contemporary research is also focused on developing novel therapeutic approaches to regulate or reverse this process, thereby reducing tissue damage resulting from excessive ECM accumulation (8–10). Various research groups have used chimeric antigen receptor (CAR) T cells to selectively eradicate activated fibroblasts as a therapeutic approach for heart failure (11). However, this method has a drawback: the indefinite survival of engineered T cells, which could be hazardous in subsequent injuries, given the crucial role fibroblast activation plays in regular wound-healing processes across diverse tissues (12). To mitigate this problem, scientists have designed a temporary antifibrotic CAR T cell therapy using nucleoside-modified mRNA technology (13).

In light of recent advancements, our research is exploring an innovative approach to liver fibrosis treatment. We are examining the use of liposomal nanoparticles (LNP), conjugated with CD163 antibodies and encapsulating FAPCAR mRNA. CD163 is macrophage-restricted, enriched in M2-polarized cells, and rapidly internalized after ligand binding. This unique LNP has the potential to specifically transfect CD163-expressing macrophages, facilitating in situ production of FAPCAR-M within the body (14), and can recognize and eliminate overactive HSCs that express FAP upon activation. This process ultimately contributes to the treatment of liver fibrosis.

## Results

### Design, Manufacturing, and Characterization of αCD163/LNP-FAPCAR.

**Fig. 1.**
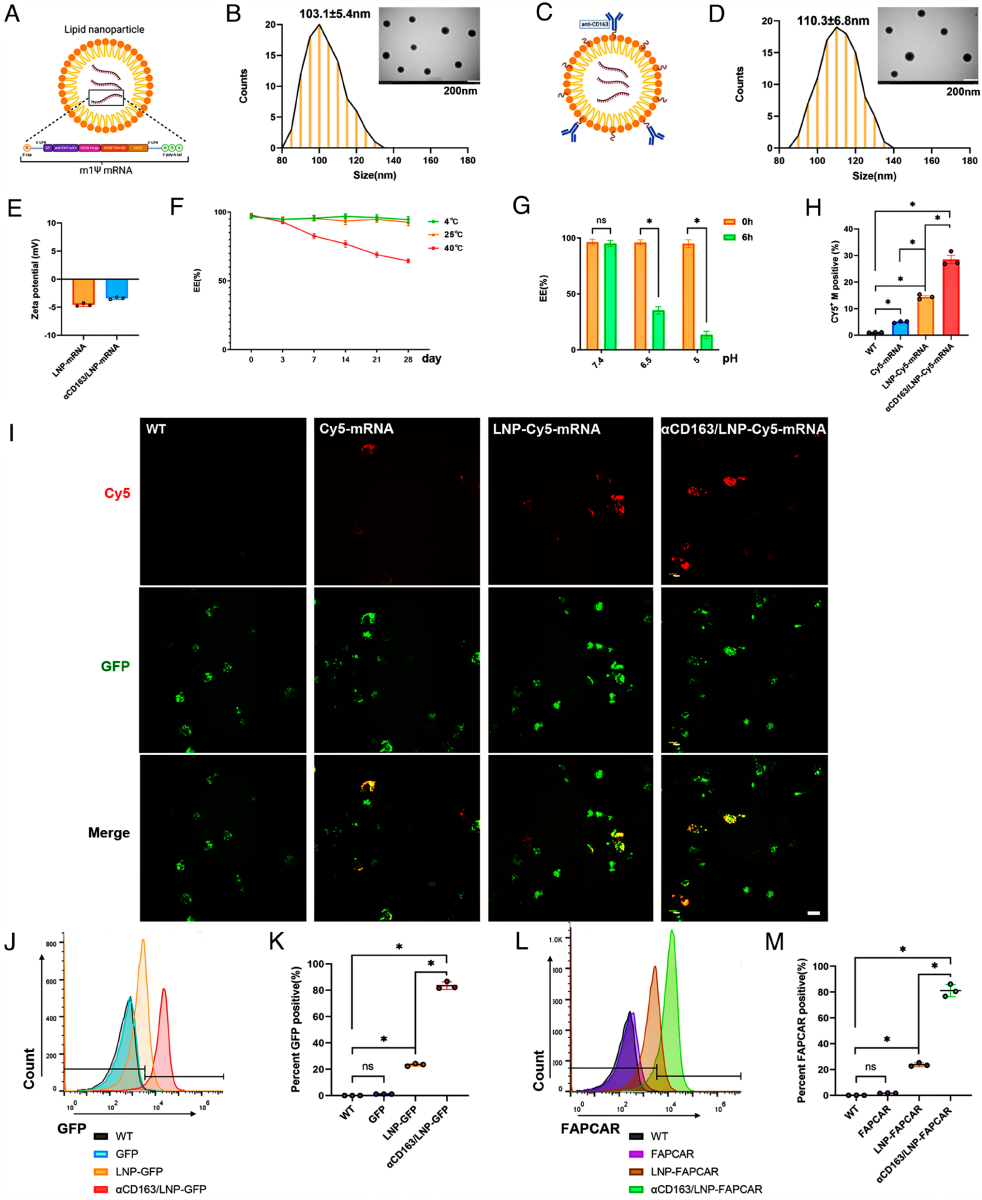
Design, fabrication, and characterization of αCD163/LNP-FAPCAR. (*A*) The schematic representation of the modification process for FAPCAR encapsulated in LNP. (*B*) A TEM image demonstrating the size distribution of LNP-FAPCAR, with over 80 particles counted. An *Inset* provided a closer view of LNP-FAPCAR. (Scale bar, 200 nm, mean ± SD.) (*C*) A structural depiction of αCD163/LNP-FAPCAR. (*D*) A TEM image demonstrating the size distribution of αCD163/LNP-FAPCAR, with over 80 particles counted. An *Inset* provided a closer view of αCD163/LNP-FAPCAR. (Scale bar, 200 nm, mean ± SD.) (*E*) An average zeta potential of LNP-FAPCAR and αCD163/LNP-FAPCAR. (*F*) Results from the mRNA analysis by RiboGreen assay quantifying the encapsulated mRNA of LNP over a time course of 28 d. (*G*) Results of mRNA analysis using the RiboGreen assay quantified the amount of mRNA encapsulated in LNPs under different pH conditions. (*H* and *I*) Statistical analysis and representative confocal fluorescence images of BMDMs transfected with mRNA, LNP-mRNA, or αCD163/LNP-mRNA. The mRNA was labeled with Cy5 (red), and BMDM expression was indicated by GFP (green fluorescence). (Scale bar, 10 μm.) (*J*) Representative GFP expression in BMDMs through flow cytometry and (*K*) the analysis of GFP expression. (*L*) Quantification of FAPCAR-positive staining in BMDMs (%) and (*M*), with analysis across biological replicates. The data were representative of three independent experiments. Differences among groups were statistically evaluated using one-way ANOVA. Significance was indicated as *P < 0.01 and non-significance as ns *P* > 0.05.

We produced a modified nucleoside-inclusive mRNA encoding an FAP-targeted CAR (*SI Appendix*, *Supplementary Materials 1*). This was subsequently encapsulated in LNPs (LNP-FAPCAR) as shown in Fig. 1*A*. The CAR architecture employed in this study incorporates the CD3ζ chain as the primary signaling domain and the CD28 intracellular domain as the costimulatory module. Before formulating LNPs, we first elucidated the functional impact of the CD28 costimulatory domain on CAR-M. Using lentiviral-mediated gene transfer, we engineered two distinct CAR-M constructs targeting FAP: αFAP-CD3ζ, which harbors the CD3ζ signaling domain alone, and αFAP-CD28-CD3ζ, which additionally encodes the CD28 intracellular costimulatory domain. To establish a robust functional validation platform, JS-1 cells were differentiated with TGF-β1, followed by FAP positivity enrichment via flow cytometry (*SI Appendix*, Fig. S1*A*). TGF-β1 induction efficiently converted approximately 80% of JS-1 cells to a FAP^+^ phenotype (*SI Appendix*, Fig. S1*B*). Coculture experiments of FAP^+^ JS-1 cells with the aforementioned CAR-M variants revealed that, relative to αFAP-CD3ζ groups, αFAP-CD28-CD3ζ groups exhibited markedly elevated phosphorylation of the p85 regulatory subunit of PI3K (*SI Appendix*, Fig. S1 *C* and *D*), concomitant with significantly enhanced phagocytic capacity toward target cells (*SI Appendix*, Fig. S1 *E* and *F*) and improved cytotoxic activity (*SI Appendix*, Fig. S1*G*).

Then TEM revealed that LNP-FAPCAR exhibited a spherical shape with a uniform particle size of 103.1 ± 5.4 nm (Fig. 1*B*) and a negative charge of −4.58 mV (Fig. 1*E*). To improve mRNA delivery to macrophages, we linked an antibody peptide targeting CD163 to the LNP surface, forming αCD163/LNP-FAPCAR (Fig. 1*C*). This modification facilitated receptor-mediated endocytosis in macrophages. The resulting αCD163/LNP-FAPCAR displayed a mean particle size of 110.3 ± 6.8 nm (Fig. 1*D*) and an average zeta potential of −3.38 mV (Fig. 1*E*). We evaluated the storage stability of the αCD163/LNP-FAPCAR-formulated mRNA under varied thermal and pH stresses. The formulation remained intact for 28 d at either 4 °C or 25 °C. The mRNA release from LNP was measured in samples stored for 1 wk after manufacturing under pressure conditions (40 °C) (Fig. 1*F*). αCD163/LNP-FAPCAR gradually discharges its mRNA cargo as the environment becomes more acidic; the lower the pH, the greater the proportion of mRNA released (Fig. 1*G*).

A red fluorescent probe (Cy5) was used to label the mRNA, and the GFP was used to label the BMDMs, revealing its internalization into the cytoplasm of BMDMs, irrespective of LNP encapsulation (Fig. 1 *H* and *I*). Notably, mRNA encapsulated in LNPs showed superior internalization efficiency. Conjugation to αCD163 further increased LNP uptake in cells, likely through receptor-induced endocytosis. We incubated BMDMs with αCD163/LNPs containing mRNA encoding FAPCAR or GFP for 48 h in vitro. Flow cytometry showed that the LNP conjugated to the αCD163 effectively delivered its mRNA cargo to target cells. Notably, over 80% of the BMDMs expressed GFP or FAPCAR upon exposure to αCD163/LNP-GFP or αCD163/LNP-FAPCAR, respectively. In contrast, unmodified LNPs delivered mRNA to fewer than 20% of target BMDMs in vitro (Fig. 1 *J*–*M*). Moreover, free mRNA without LNP encapsulation expressed the target protein poorly, likely due to inefficient escape from lysosomes.

### In Vitro Functional Evaluation of αCD163/LNP-FAPCAR.

**Fig. 2.**
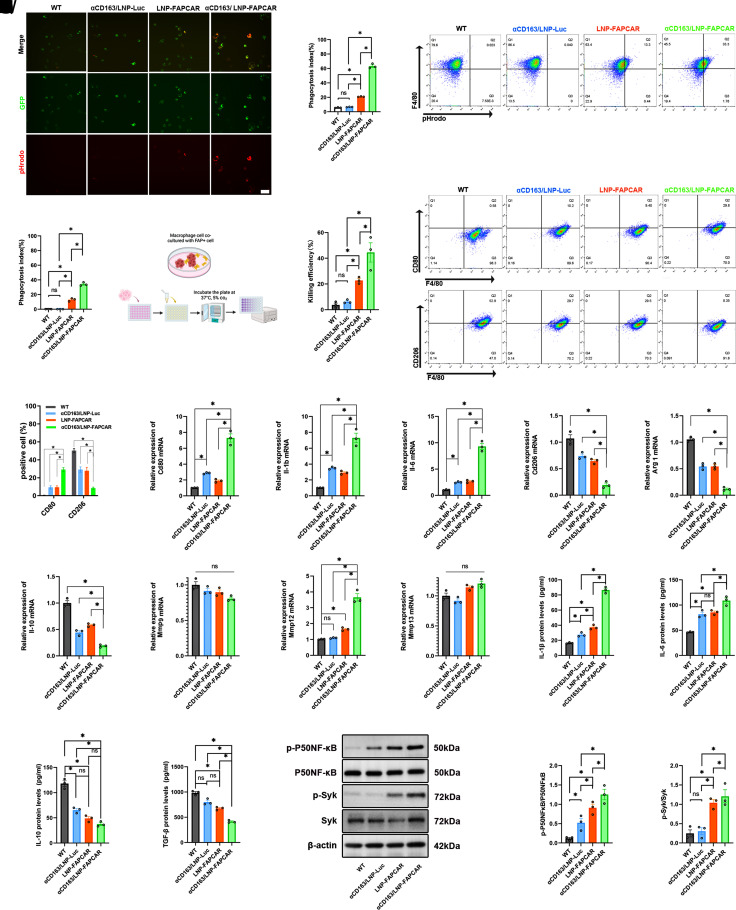
In vitro functional assessment of αCD163/LNP-FAPCAR. (*A*) Confocal microscopy images showed phagocytosis of FAP^+^JS-1 cells by BMDMs treated with various formulations. The JS-1 cells were labeled with pHrodo, and BMDMs were labeled with GFP (green fluorescence). Red fluorescence denoted specific phagocytosis of BMDMs on JS-1 cells labeled with pHrodo. (Scale bar, 10 μm.) (*B*) Quantitative analysis of the phagocytic index in BMDMs. (*C* and *D*) Flow cytometry was used to assess the phagocytic activity of BMDM cells in response to various formulations using pHrodo-labeled FAP^+^JS-1 cells and the statistical analysis. (*E*) A schematic diagram was provided to illustrate the cell-killing experiment. (*F*) The cytotoxicity of BMDMs against target FAP^+^JS-1 cells. (*G* and *H*) Flow cytometry analysis of CD80 and CD206 expression in the screened BMDMs and the analysis. Gate strategy: exclude debris/dead cells (*Left* side) through FSC (cell size) and SSC (particle size). BMDMs with unincubated flow cytometry antibodies serving as a negative control gate method. (*I*–*Q*) The mRNA relative expression of *Cd80, Il-1β, IL-6, Cd206, Arg1, Il-10, Mmp9*, *Mmp12,* and *Mmp13* in screened BMDMs. (*R*–*U*) The protein levels of IL-1β, IL-6, IL-10, and TGF-β1 of the supernatant of the screened BMDMs. (*V*–*X*) The relative protein expression level of p-P50NF-κB/P50NF-κB and p-Syk/Syk of screened BMDMs. (n = 3). Differences among groups were statistically evaluated using one-way ANOVA. Significance was indicated as *P < 0.01 and non-significance as ns *P* > 0.05.

Subsequent to treatment, we evaluated functional alterations in BMDMs. Both αCD163/LNP-FAPCAR and LNP-FAPCAR treatments significantly enhanced the phagocytic rates of BMDMs. Notably, the αCD163/LNP-FAPCAR treatment induced a phagocytic rate more than ten times that of the WT group and approximately three times that of the LNP-FAPCAR group. Contrarily, the αCD163/LNP-Luc group did not exhibit increased phagocytic efficiency, indicating that FAPCAR expression was crucial for enhancing macrophage-mediated phagocytosis of FAP^+^JS-1 cells (Fig. 2 *A*–*D*). Moreover, we validated the cytotoxicity of FAPCAR-expressing BMDMs against target cells. By transfecting target cells with luciferase, we quantified the killing effect, and the results demonstrated that the CAR-M derived from αCD163/LNP-FAPCAR significantly eliminated the FAP^+^JS-1 target cells in vitro (Fig. 2 *E* and *F*). Subsequently, we examined the polarization of BMDMs screened in the FAP^+^JS-1 cell coculture system (*SI Appendix*, Fig. S1*H*). The results showed that BMDMs, especially those treated with αCD163/LNP-FAPCAR, significantly promoted the expression of the M1 marker CD80 (Fig. 2 *G*–*I*), as well as mRNA (Fig. 2 *J* and *K*) and protein expression of IL-1β and IL-6 (Fig. 2 *R* and *S*). Meanwhile, the αCD163/LNP-FAPCAR group reduced the expression of the M2 marker CD206 (Fig. 2 *G* and *H*), and the mRNA levels of *Cd206*, *Arg1*, and *Il-10* (Fig. 2 *L*–*N*), as well as the protein levels of IL-10 and TGF-β1 (Fig. 2 *T* and *U*). Notably, the αCD163/LNP-FAPCAR group could induce higher *Mmp12* gene expression, and no significant effect on the gene expression of *Mmp9* and *Mmp13* (Fig. 2 *O*–*Q*). Pretreatment with the macrophage-specific phagocytosis inhibitor Cytochalasin D abrogated the MMP12 expression in CAR-M, establishing phagocytosis as an obligate upstream event governing MMP12 transcriptional induction (*SI Appendix*, Fig. S2 *A*–*C*). Specific CAR-mediated target recognition elicited proinflammatory reprogramming of BMDMs. Mechanistically, immunoblot analyses demonstrated that CAR engagement with target cells directly activated the Syk/NF-κB signaling axis, driving macrophage polarization toward a proinflammatory phenotype (Fig. 2 *V*–*X*). In the absence of target cells, LNP formulation alone was sufficient to activate the Syk/Myd88/NF-κB signaling axis, indicating that LNPs may elicit macrophage inflammatory responses through pattern recognition receptor (PRR)-mediated, antigen-independent mechanisms (*SI Appendix*, Fig. S2 *D*–*H*). Upon engagement with the target antigen FAP, the immunoreceptor tyrosine-based activation motif (ITAM) within the CAR scaffold recruited and phosphorylated Syk, thereby amplifying the Syk/Myd88/NF-κB signaling cascade (*SI Appendix*, Fig. S2 *I*–*M*). Pharmacological inhibition of Syk (BAY 61-3606) abrogated downstream Myd88/NF-κB activation (*SI Appendix*, Fig. S2 *N*–*R*), confirming that FAP-directed recognition specifically engaged the Syk/MyD88/NF-κB pathway to potentiate proinflammatory reprogramming of BMDMs.

Another, in vitro simulated fibrosis environment (*SI Appendix*, Fig. S3*A*), αCD163/LNP-FAPCAR can maintain the killing effect of BMDMs on target cells (*SI Appendix*, Fig. S3*B*). Furthermore, this intervention could attenuate the profibrotic phenotype of residual JS-1 cells that evaded cytotoxic elimination (*SI Appendix*, Fig. S3 *C*–*G*) and still reprogram BMDMs into a proinflammatory phenotype (*SI Appendix*, Fig. S3 *H*–*O*).

In addition, we engineered human-derived FAPCAR mRNA (h-αCD163/LNP-FAPCAR). First, the Flow cytometry showed that over 70% of the macrophages (THP-1 cells induced with PMA) expressed FAPCAR upon exposure to h-αCD163/LNP-FAPCAR. In contrast, unmodified LNPs could only deliver mRNA to less than 20% of the target macrophages in vitro (*SI Appendix*, Fig. S4 *A* and *B*). Then we examined the impact of various treatments on macrophages’ phagocytosis of LX-2 cells in a fibrosis-mimicking environment. The h-αCD163/LNP-FAPCAR treatments significantly enhanced macrophage phagocytic rates (*SI Appendix*, Fig. S4 *C*–*F*) and cytotoxicity (*SI Appendix*, Fig. S4*G*), and h-αCD163/LNP-FAPCAR can still reprogram macrophages into a proinflammatory phenotype (*SI Appendix*, Fig. S4 *H*–*O*).

### Delivery of αCD163/LNP-FAPCAR to Macrophages in Fibrotic Liver via Targeted Approach.

We administered LNP carrying either Photinus pyralis luciferase (LNP-Luc or αCD163/LNP-Luc) or ZsGreen (LNP-ZsGreen or αCD163/LNP-ZsGreen) mRNA into the fibrotic mice induced by CCL4. In vivo bioluminescence imaging revealed that both unmodified LNP-Luc and αCD163-modified LNP-Luc formulations achieved peak fluorescence intensity at 8 h postinjection, with signal attenuation becoming evident after 24 h. Notably, unmodified LNP-Luc exhibited systemic biodistribution, potentially attributable to enhanced lysosomal escape characteristics, whereas αCD163/LNP-Luc demonstrated selective hepatic enrichment (*SI Appendix*, Fig. S5*A*). Ex vivo organ imaging corroborated sustained accumulation of αCD163/LNP-Luc in liver and splenic tissues (*SI Appendix*, Fig. S5*B*). ZsGreen mRNA tracking studies demonstrated prolonged hepatic fluorescence persistence in the αCD163/LNP-ZsGreen cohort (sustained through day 14). In contrast, the unmodified LNP-ZsGreen group exhibited rapid signal decline by day 7 and complete clearance by day 14 (*SI Appendix*, Fig. S5 *C* and *D*). Pharmacokinetic profiling revealed that circulating mRNA in the LNP-ZsGreen group peaked at 2 h and was fully cleared within 24 h, whereas αCD163/LNP-ZsGreen exhibited delayed clearance kinetics extending to 48 h (*SI Appendix*, Fig. S5*E*). Then, the antibody-mediated targeted cellular uptake was assessed on the 7th day postinjection (*SI Appendix*, Fig. S5*F*). In the αCD163/LNP-ZsGreen cohort, surface CD163 expression was markedly diminished, with minimal overlap between ZsGreen^+^ and CD163^+^ populations. Antibody-mediated CD163 internalization constitutes the principal mechanism underlying this surface downregulation. Conversely, unmodified LNP operates independently of CD163-mediated uptake, resulting in substantial ZsGreen/CD163 colocalization, thereby indirectly corroborating the targeting function of conjugated antibodies (*SI Appendix*, Fig. S5*G*). Flow cytometric quantification of ZsGreen^+^/CD163^+^ macrophages further validated these observations: The LNP-ZsGreen group exhibited a high frequency of CD163^+^ macrophages, with nearly all ZsGreen^+^ cells coexpressing CD163. In stark contrast, the αCD163/LNP-ZsGreen cohort displayed reduced CD163 positivity and a markedly diminished proportion of ZsGreen^+^/CD163^+^ double-positive macrophages (*SI Appendix*, Fig. S5 *H*–*J*). In colocalization studies, LNP exhibited specific expression of ZsGreen protein in macrophages of fibrotic mice, with no detectable expression in other hepatic cell types, whereas unmodified LNP predominantly transduced hepatocytes (*SI Appendix*, Fig. S5 *K* and *L*). These findings established that CD163 antibody-LNP mRNA formulations confer macrophage-specific delivery with enhanced targeting precision and transfection efficiency in hepatic tissue. Furthermore, both liver fibrotic (CCL4+αCD163/LNP-FAPCAR group) and normal mice (oil + αCD163/LNP-FAPCAR group) that received LNP injections exhibited FAPCAR^+^ macrophages, signifying successful FAPCAR mRNA transduction; no FAPCAR signal was detected in CCL4+PBS controls (*SI Appendix*, Fig. S5 *M* and *N*). In the CCL4+αCD163/LNP-FAPCAR group, an accumulation of FAPCAR^+^ macrophages was observed in areas populated by HSCs (Desmin^+^), and a phenomenon absent in the fibrotic mice (CCL4 + PBS group). Conversely, the oil + αCD163/LNP-FAPCAR group exhibited FAPCAR^+^ macrophages, but without any accumulation in Desmin^+^ regions (*SI Appendix*, Fig. S5*O*). We then evaluated the nanoparticles’ biosafety. There were no significant changes in the main organs (*SI Appendix*, Fig. S6*A*), body mass (*SI Appendix*, Fig. S6*B*), or hematological and biochemical parameters (*SI Appendix*, Fig. S6*C*). Collectively, these findings establish that this targeted delivery paradigm enhances mRNA transfection efficiency in designated cellular compartments while concomitantly mitigating off-target liabilities arising from promiscuous uptake, thereby augmenting the therapeutic index and furnishing a precision medicine platform for hepatic fibrosis intervention.

### The Administration of αCD163/LNP-FAPCAR Effectively Mitigated Liver Damage in a Mouse Model of Hepatic Fibrosis.

**Fig. 3.**
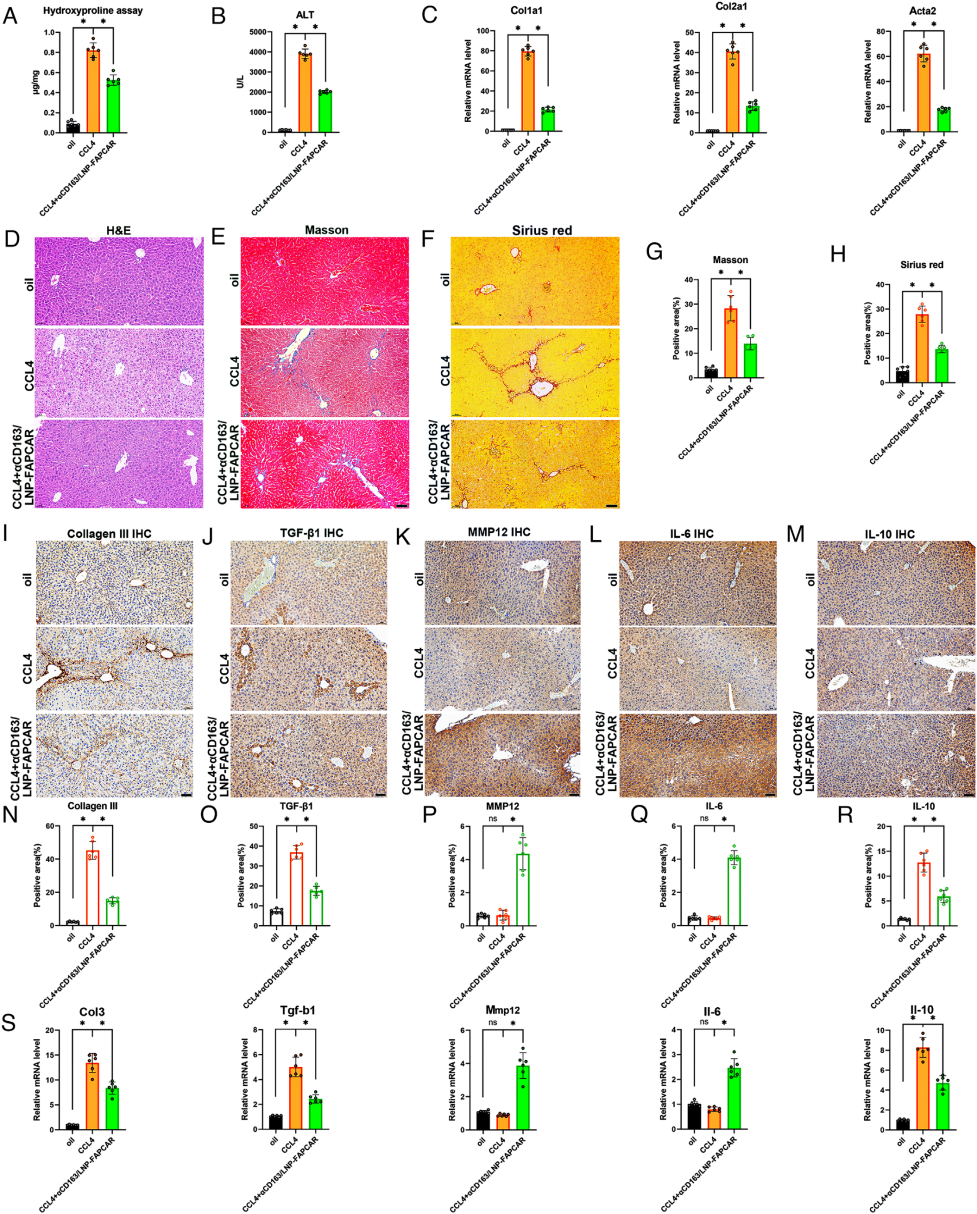
αCD163/LNP-FAPCAR delivery mitigated liver damage in mice. (*A*) Hydroxyproline quantification of the mice. (*B*) The serum ALT level determination. (*C*) The qPCR analysis of *col1a1*, *col2a1*, and *acta2* in liver tissues from the mice. (*D*–*H*) H&E, Sirius red, and Masson’s trichrome staining. (Scale bar, 50 μm.) with corresponding quantitative analysis. (*I*–*R*) The IHC staining of Collagen III, TGF-β1, MMP12, IL-6, and IL-10. (Scale bar, 50 μm.) with corresponding quantitative analysis. (*S*) The qPCR analysis of *Col3, Tgf-b1, Mmp12, Il-6,* and *Il-10* in the liver of the mice. (n = 6). Differences among groups were statistically evaluated using one-way ANOVA. Significance was indicated as *P < 0.01 and non-significance as ns *P* > 0.05.

To explore whether αCD163/LNP-FAPCAR can improve liver function in vivo, we detected the extent of liver injury by evaluating the hydroxyproline (Fig. 3*A*) and ALT (Fig. 3*B*) content and the level of fibrosis-related genes through qRT-PCR analysis (Fig. 3*C*). The results showed that there was a high level of expression of the targets above in the CCL4 group. The treatment of αCD163/LNP-FAPCAR inhibited this. Meanwhile, we observed that mice treated with αCD163/LNP-FAPCAR could significantly alleviate liver fibrosis as assessed by histology, Sirius red staining, and Masson’s trichrome staining (Fig. 3 *D*–*H*), and by fibrosis markers Collagen III and TGF-β1 (Fig. 3 *I*, *J*, *N*, and *O*). Furthermore, we found that MMP12 and IL-6 expression were much higher in the treatment group (Fig. 3 *K*, *L, P*, and *Q*). Another, IL-10 was significantly inhibited (Fig. 3 *M* and *R*). And the gene expression levels of *Col3, Tgf-b1*, *Mmp12, Il-6*, and *Il-10* were identical to the protein levels (Fig. 3*S*). To elucidate the mechanistic basis of antibody-conjugated LNP-mediated therapeutic efficacy in hepatic fibrosis, we conducted a comparative analysis of three intervention cohorts: αCD163/LNP-Luc (luciferase mRNA control), unmodified LNP-FAPCAR (FAPCAR mRNA without targeting moiety), and αCD163/LNP-FAPCAR (FAPCAR mRNA with CD163-directed antibody conjugation) (*SI Appendix*, Fig. S7 *A*–*O*). The αCD163/LNP-Luc cohort exhibited no appreciable therapeutic benefit. In contrast, the LNP-FAPCAR group demonstrated modest improvements in select fibrosis-associated biomarkers, indicating that hepatic accumulation of FAPCAR-M conferred partial antifibrotic activity. Notably, the αCD163/LNP-FAPCAR cohort achieved the most pronounced fibrosis regression. IHC analyses of hepatic tissue further validated that αCD163/LNP-FAPCAR treatment enabled targeted elimination of FAP^+^ cells (*SI Appendix*, Fig. S7 *P* and *Q*), establishing FAPCAR-M as the critical effector population driving therapeutic resolution of liver fibrosis. At the same time, we established mouse models of liver fibrosis induced by BDL and MCD diet to test the therapeutic efficiency of αCD163/LNP-FAPCAR. The results showed that treatment with αCD163/LNP-FAPCAR effectively alleviates BDL- and MCD-diet-induced liver fibrosis in mice (*SI Appendix*, Figs. S8 and S9), reduces collagen III expression and TGF-β1 levels, and increases MMP12 and IL-6 expression, which contribute to liver fibrosis repair. These analyses collectively demonstrated that αCD163/LNP-FAPCAR effectively mitigated liver damage and fibrosis in mice.

### αCD163/LNP-FAPCAR Suppressed Liver Fibrosis via Facilitating Liver Tissue Repair and Inhibiting HSC Activation.

**Fig. 4.**
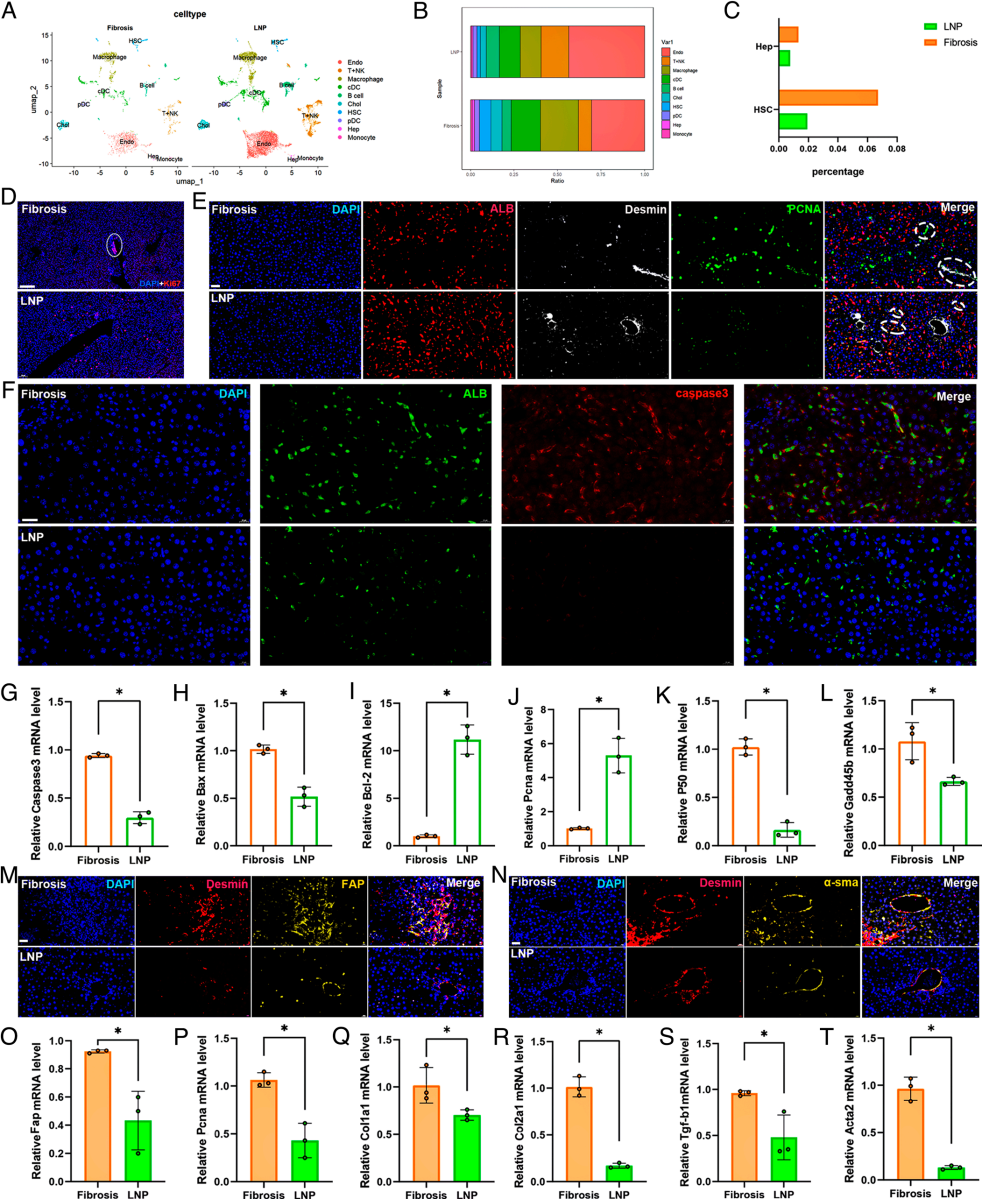
αCD163/LNP-FAPCAR enhanced the liver tissue repair and suppressed HSC activation. (*A*) UMAP illustrated cell clustering in both the fibrosis (CCL4-induced fibrosis mice model) and the LNP (αCD163/LNP-FAPCAR) treatment groups. (*B* and *C*) The proportion of cells within Heps, HSCs, macrophages, and non-parenchymal cell clusters. (*D*) The Ki67 fluorescent staining in the liver tissues. (Scale bar, 100 μm.) (*E*) The IF confocal microscopy of ALB^+^ (green), Desmin^+^ (red), PCNA^+^ (yellow), and DAPI in the liver tissues. (Scale bar, 20 μm.) (*F*) The IF confocal microscopy of ALB^+^ (green), caspase3^+^ (red), and DAPI in the liver tissues. (Scale bar, 20 μm.) (*G*–*L*) The qPCR analysis of *Caspase3*, *Bax*, *Bcl-2*, *Pcna*, and *P50*, *Gadd45b* of the extracted primary Heps from the mouse liver. (*M*) The fluorescence confocal microscopy of Desmin^+^ (red), FAP^+^ (yellow), and DAPI in the liver tissues. (Scale bar, 20 μm.) (*N*) The fluorescence confocal microscopy of Desmin^+^ (red), α-sma^+^ (yellow), and DAPI in the liver tissues. (Scale bar, 20 μm.) (*O*–*T*) The qPCR analysis of *Fap*, *Pcna, Col1a1, Col2a1, Tgf-b1,* and *Acta2* of the extracted HSCs from the mouse liver. (n = 3). Differences among groups were statistically evaluated using one-way ANOVA. Significance was indicated as *P < 0.01 and non-significance as ns *P* > 0.05.

To further elucidate the molecular and cellular alterations induced by αCD163/LNP-FAPCAR, liver tissues were collected from fibrotic model mice (Fibrosis group) and those treated with αCD163/LNP-FAPCAR (LNP group) for scRNA-seq. This method identified 11 unique cell clusters (cluster 0 to 10) (*SI Appendix*, Fig. S10 *A* and *B*). We examined gene expression profiles of various cell clusters. We identified multiple liver cell populations, including endothelial cells (Endo), B cells, hepatocytes (Heps), HSCs, macrophages, dendritic cells (pDC, cDC), monocytes, and cholangiocytes (Chol) (Fig. 4*A* and *SI Appendix*, Fig. S10 *C* and *D*). No unique cell clusters were found in either group when comparing clustering patterns (*SI Appendix*, Fig. S10*E*). We also determined and compared the proportions of each cell type in the samples (Fig. 4*B*). We found that the LNP group had a lower proportion of HSCs than the Fibrosis group. It was worth noting that the proportion of Heps had also decreased slightly (Fig. 4*C*). Therefore, we compared the transcriptomic differences in Heps between the Fibrosis and LNP groups (*SI Appendix*, Fig. S10*F*) and conducted GO enrichment analysis of the DEGs in Heps. The results showed that the DEGs were mainly enriched in GO terms such as regulation of actin cytoskeleton organization, regulation of cytoskeleton organization, regulation of organelle organization, reproductive process, reproduction, meiotic cell cycle, etc (*SI Appendix*, Fig. S10*G*). Compared with the Fibrosis group, the LNP group promoted proliferation-related biological processes in Heps, as indicated by GSEA of GO terms (*SI Appendix*, Fig. S10*H*). These results indicated that although the proportion of Heps in the LNP group was lower, those Heps showed greater proliferative capacity. To verify the Heps results via enrichment analysis, we assessed Ki67 staining in liver tissue samples from both groups. In fibrotic liver tissues, Ki67 signals predominantly congregated around endothelial cells and the HSC aggregation region, with minimal Ki67^+^ signals detected distally from endothelial cells. Conversely, following αCD163/LNP-CAR treatment, Ki67 signals appeared more frequently distant from endothelial cells (Fig. 4*D*). To further investigate the cell proliferation pattern of liver tissues, we performed fluorescence staining of liver tissues with PCNA [PCNA is often used as a proliferation or regeneration marker because it is only expressed in mitotic cells (15)], ALB, and Desmin. We found that more Heps (ALB^+^) in the LNP group expressed PCNA, whereas HSCs (Desmin^+^) showed almost no PCNA expression. In the Fibrosis group, PCNA expression was significantly increased in HSCs and was almost absent in Heps (Fig. 4*E*). Furthermore, we detected apoptosis in Heps in both groups. The IF results showed that the apoptosis of Heps in the LNP group was significantly lower (Fig. 4*F*). Also, we verified that the LNP treatment could inhibit the genes of caspase3 and bax and promote bcl-2 and pcna expression in the extracted primary Heps from the mouse liver through qPCR analysis (Fig. 4 *G*–*J*). And we found that the LNP treatment could inhibit p50 and gadd45b, which are involved in the NF-κB pathways and mainly mediate the release of inflammatory factors and cellular activity (*SI Appendix*, Fig. S10 *I* and *J*). We verified that the LNP treatment inhibited the expression of p50 and gadd45b in primary Heps (Fig. 4 *K* and *L*). These results suggested that the LNP treatment promoted Heps proliferation by regulating the p50 and gadd45b signaling pathways, whereas, relative to the Fibrosis group, the LNP treatment cohort exhibited a modest reduction in the proportion of Heps, presumably reflecting the clearance of apoptotic Heps.

We also demonstrated the clearance effect of the LNP treatment on activated HSCs through fluorescence staining. As shown in Fig. 4 *M* and *N*, the treatment with LNP reduced the expression of FAP^+^HSCs and α-sma^+^HSCs. Furthermore, we verified that the LNP treatment could inhibit the expression of fap, pcna, col1a1, col2a1, tgf-b1, and acta2 in primary HSCs isolated from the mouse liver (Fig. 4 *O*–T**). Collectively, these data indicate that αCD163/LNP-FAPCAR delivery can mitigate liver fibrosis by inhibiting HSCs activation, enhancing hepatic regeneration, and facilitating tissue repair.

### αCD163/LNP-FAPCAR Enhanced MMP12^+^ Scar Clearance Phenotype by Reprogramming Macrophage Subpopulations to Alleviate Liver Fibrosis.

**Fig. 5.**
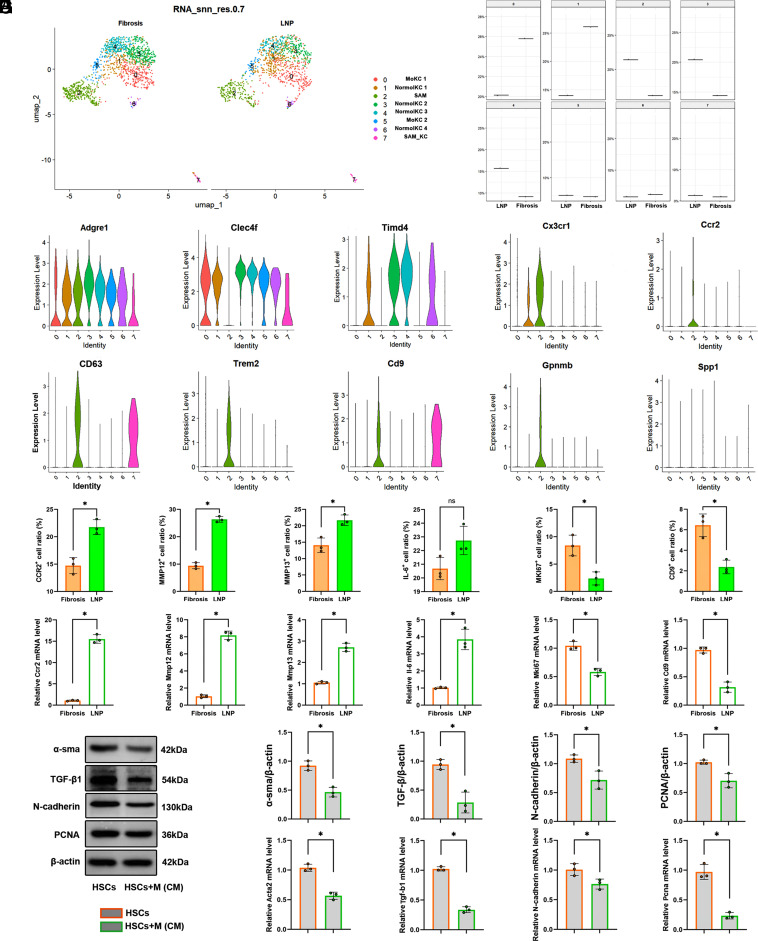
Treatment with αCD163/LNP-FAPCAR effectively skewed their polarization toward a proreparative phenotype. (*A*) UMAP illustrates macrophages clustering in both the fibrosis and LNP groups. (*B*) Intergroup comparison of the proportion of cells in each cluster. (*C*) Vlnplot analysis of the marker gene expression in various macrophage clusters. (*D*) Flow cytometry analysis of CCR2, MMP12, MMP13, IL-6, MKI67, and CD9 expression in primary macrophages from mouse liver and across biological replicates. (*E*) The qPCR analysis of *Ccr2*, *Mmp12*, *Mmp13*, *Il-6*, *Mki67,* and *Cd9* expression of the extracted primary macrophages. (*F* and *G*) The relative protein expression levels of α-SMA, TGF-β1, N-cadherin, and PCNA in HSCs were analyzed by western blot. (*H*) The qPCR analysis of *Acta2*, *Tgf-b1*, *N-cadherin,* and *Pcna* expression of HSCs. (n = 3). Differences among groups were statistically evaluated using one-way ANOVA. Significance was indicated as *P < 0.01 and non-significance as ns *P* > 0.05.

Then we observed a significant difference in the proportion of macrophages among the samples, with the LNP group showing a lower proportion (*SI Appendix*, Fig. S11*A*). Following αCD163/LNP-FAPCAR intervention, the hepatic macrophage compartment underwent selective depletion, prompting investigation into the regulatory dynamics of macrophages in fibrogenesis. The scRNA-seq results revealed the presence of FAP^+^ cells within the macrophage subset in fibrotic livers, with αCD163/LNP-FAPCAR treatment markedly attenuating the amount of FAP^+^ macrophages; conversely, transcriptional levels of Acta2 and Col1a1 remained statistically unchanged between cohorts (*SI Appendix*, Fig. S11 *B–G*). IF colocalization studies corroborated these findings, fibrotic liver tissue exhibited FAP/F4/80 double-positive macrophages, whereas such populations were virtually eradicated following αCD163/LNP-FAPCAR administration (*SI Appendix*, Fig. S11*H*). Immunophenotyping subsequently identified FAP-specific T cell populations at the splenic red pulp–white pulp interface in the treated mice (*SI Appendix*, Fig. S11*I*). To interrogate the functional competence of these T cells, splenic T lymphocytes were isolated and cocultured ex vivo with TGF-β1-induced macrophages (*SI Appendix*, Fig. S11*J*). Notably, T cells derived exclusively from the αCD163/LNP-FAPCAR cohort elicited antigen-specific cytotoxicity against FAP^+^ macrophages (*SI Appendix*, Fig. S11*K*). To delineate the immunomodulatory effects of αCD163/LNP-FAPCAR on hepatic macrophage heterogeneity, we conducted comparative transcriptomic profiling of macrophage subpopulations across treatment cohorts (*SI Appendix*, Fig. S12*A*), followed by GSEA (*SI Appendix*, Fig. S12*B*). Notably, antigen presentation–associated pathways were significantly upregulated within the macrophage compartment of the αCD163/LNP-FAPCAR group (*SI Appendix*, Fig. S12*C*), substantiating the generation of functionally competent FAPCAR-M capable of target cell phagocytosis and enhanced memory immune responses. Concomitantly, lipid metabolism and atherosclerosis-related pathways showed significant enrichment (*SI Appendix*, Fig. S12*D*), whereas NF-κB signaling, mediated by Toll-like receptor activation and downstream IL-6 release, characterized the LNP-only cohort (*SI Appendix*, Fig. S12*E*). In a comparative subgroup analysis of macrophages, we compared the two groups. Our subgroup analysis of macrophages showed that they were divided into 8 clusters (cluster 0 to 7) (Fig. 5*A*). We manually annotated these macrophage clusters. We classified these clusters using macrophage markers Adgre1 (F4/80), monocyte markers Ccr2 and Cx3cr1 (16), as well as Kupffer cells (KC) markers Clec4f; meanwhile, Timd4 can serve as a key phenotype marker, effectively distinguishing monocyte-derived Kupffer cells (MoKC) from resident Kupffer cells (NormolKC). It is noteworthy that scar-associated macrophages (SAM) and lipid-associated macrophages (LAM) coexist in the fibrotic microenvironment. SAM exhibits characteristic expression of surface markers such as Cd63, Trem2, Cd9, and GpnmB, while LAM has a similar gene expression profile but can achieve phenotypic differentiation through specific high expression of osteopontin (SPP1) (17). Among them, Clusters (0, 5) were MoKC derived from Timd4^−^Clec4f^+^ macrophages, while clusters (1, 3, 4, 6) were general NormolKC derived from Timd4^+^Clec4f^+^ macrophages. Given negligible SPP1 expression across all clusters, the analyzed macrophage landscape was predominantly enriched for SAM-like states. And subgroup identification was achieved by specific expression of Clec4f, with cluster 2 clearly identified as classical SAM, while cluster 7 exhibited SAM-like KC (SAM_KC) (Fig. 5 *A* and *C*). We compared the proportion of cells in different clusters between the two groups. We found that, compared to the Fibrosis group, the LNP group had a higher proportion of clusters (2–4), while the other clusters (0, 1) were relatively lower (Fig. 5*B*). Among them, the expression of Trem2, Mmp12, and Mmp13 associated with the elimination of scar tissue was higher specific to cluster 2, the expression of inflammatory factor IL-6 was higher in cluster (1, 4, 5), the Mki67 associated with proliferation specific to cluster 6 was expressed higher (*SI Appendix*, Fig. S12*F*). Critically, within the SAM lineage (cluster 2), MMP12 and MMP13 expression were significantly potentiated in the LNP group compared to fibrotic counterparts. And the flow cytometric validation of hepatic F4/80^+^ macrophages (*SI Appendix*, Fig. S12*G*) confirmed elevated frequencies of CCR2^+^/MMP12^+^/MMP13^+^/IL-6^+^ subsets, with particularly pronounced enrichment of MMP12^+^ macrophages, alongside reduced MKI67^+^/CD9^+^ populations relative to fibrotic controls (Fig. 5*D*). These protein-level observations aligned with transcriptional data (Fig. 5*E*). Then, we established the coculture system of the macrophages and TGF-β1-induced HSCs (*SI Appendix*, Fig. S12*H*). The results demonstrated that, compared with HSCs induced solely by TGF-β1, HSCs exposed to MMP12^+^macrophage-conditioned medium (CM) expressed fewer fibrosis-related proteins and mRNAs (Fig. 5 *F*–*H*). The results indicated that LNP treatment altered the proportions of different macrophage cluster subtypes, especially increasing MMP12 expression in macrophage subtypes involved in scar elimination. Finally, to explore the therapeutic potential of targeted FAP treatment for clinical applications, we collected liver fibrosis tissue from patients with different etiologies and performed IHC staining for FAP. The results showed that, compared with nonfibrotic liver tissues, liver fibrosis caused by various etiologies was associated with increased FAP expression (*SI Appendix*, Fig. S12*I*).

## Discussion

This study presents a method for targeting HSCs in liver fibrosis using CD163 antibody-conjugated LNPs containing FAPCAR mRNA. By capitalizing on the specific expression of CD163 on macrophages, these LNPs can selectively transfect and induce CAR expression in liver macrophages, driving proinflammatory reprogramming of macrophages, which may occur by triggering Syk and NF-κB phosphorylation cascades downstream of the CAR. Additionally, these LNPs increased the expression of MMP12 in macrophages (Figs. 1 and 2). And the MMP12 induction is contingent upon macrophage-mediated phagocytosis (*SI Appendix*, Fig. S2*A*). The resulting CAR-M can recognize and eliminate FAP^+^ HSCs, crucial contributors to liver fibrosis. This approach offers several benefits compared to conventional treatment strategies. First, it allows selective elimination of profibrotic cells while preserving healthy Heps by targeting FAP^+^ HSCs. Second, generating CAR-M cells in situ within the liver microenvironment may enhance efficacy and safety compared with systemic drug delivery therapy (*SI Appendix*, Figs. S5 and S6).

Notably, the FAPCAR architecture employed in this study incorporates the classical CD28 costimulatory domain paired with the CD3ζ signaling domain-a configuration conferring dual functional advantages. The CD28 domain specifically promotes phosphorylation of the PI3K regulatory subunit p85 via PI3K pathway activation, thereby markedly enhancing phagocytic competence (18–21). Concurrently, the CD3ζ primary signaling domain generates activation cues analogous to those of conventional immune receptors (e.g., FcγRI) in macrophages (22), with its ITAM efficiently recruiting Syk kinase to initiate downstream signaling cascades (23). This classical domain pairing endows macrophages with superior phagocytic capacity while simultaneously reprogramming cellular metabolic states through synergistic signal integration (*SI Appendix*, Fig. S1).

We employed a nonconventional, noncommercially available LNP formulation in this study. Unmodified LNP (lacking CD163 antibody conjugation) exhibited substantial CAR expression in the systemic circulation-an observation attributable to two key formulation optimizations. First, the elevated DSPC content (30 mol%) significantly potentiated LNP-monocyte-derived cell interactions (24), facilitating uptake by circulating monocytes. Second, the ionizable lipid heptadecan-9-yl 8-[(2-hydroxyethyl) (8-(nonyloxy)-8-oxooctyl) amino] octanoate substantially enhances endosomal escape efficiency (25), enabling the effective cytoplasmic mRNA release. These “passive targeting” characteristics result in the widespread CAR expression among circulating immune cells. However, the surface conjugation of CD163 antibodies fundamentally remodels LNP biodistribution. The antibody mediates receptor-directed delivery of LNP to hepatic and splenic macrophages: αCD163 binds to cognate CD163 receptors, triggering clathrin-dependent endocytosis, thereby allowing LNP to bypass lysosomal degradation and enter the intracellular trafficking network directly. This active targeting mechanism not only markedly enhances intrahepatic transfection efficiency but also restricts the CAR expression to the lesion microenvironment, thereby precluding systemic off-target liabilities. The present findings established that CD163 antibody-conjugated LNPs exhibited markedly superior target protein accumulation kinetics and prolonged expression persistence in fibrotic hepatic tissues compared to unmodified LNP formulations (*SI Appendix*, Fig. S5 *A*–*D*). This enhanced performance may be partially attributable to antibody-mediated alterations in LNP physicochemical properties (such as surface charge or hydrodynamic diameter). However, the predominant determinant likely resided in differential cellular targeting. As terminally differentiated cells, macrophages exhibit low proliferation rates that effectively prevent dilution of transgenic expression caused by cell division (26), thereby maintaining stable expression of the FAPCAR protein for up to 14 d. The protein of interest was exclusively expressed in macrophages, thereby mitigating the risk of off-target effects.

Despite the potential for expression of αCD163/LNP-FAPCAR in healthy mouse tissues, the absence of FAP precluded the functionality of transfected FAPCAR-M in these tissues (*SI Appendix*, Fig. S5*O*). This suggested a theoretically safer approach compared to other mRNA delivery strategies (27, 28). Single-cell sequencing facilitated a thorough understanding of this treatment approach. αCD163/LNP-FAPCAR mitigated excessive ECM deposition by phagocytosing activated HSCs, fostering the regeneration of damaged liver parenchymal cells by adopting a tissue repair phenotype post target cell elimination. Normally, p50 and gadd45b act as antiapoptotic proteins that promote cell proliferation (29, 30). However, our study found that after treatment, the expression of p50 and gadd45b, as well as caspase3 and bax (the markers of apoptosis), decreased in primary Heps, whereas ki67 expression increased (Fig. 4 *G*–*L*). In research on liver-related diseases, the role of Gadd45b expression remains controversial (31). The high expression of gadd45b in liver cancers can promote cell apoptosis by activating the p38/JNK signaling pathway (26). It has also been found that high expression of gadd45b can promote cell apoptosis by inducing TGF-β1 (32). This was consistent with our research findings, which suggested that inflammatory factors, such as TGF-β1 secreted during liver fibrosis, may promote apoptosis by increasing gadd45b expression. LNP treatment can inhibit gadd45b expression in the liver of fibrotic mice, thereby suppressing hepatocyte apoptosis. Our findings further demonstrated that αCD163/LNP-FAPCAR administration globally diminished the hepatic macrophage compartment, attributable to the selective depletion of specific macrophage subpopulations. The LNP platform enabled the sustained FAPCAR expression in macrophages, facilitating the target cell engulfment and subsequent antigen presentation. This signaling cascade elicited the activation of FAP-specific T cell populations residing at the splenic red pulp-white pulp interface, which subsequently orchestrated selective cytotoxicity against FAP-expressing macrophage subsets that had been induced by TGF-β1 and other profibrotic cues within the fibrotic microenvironment (*SI Appendix*, Fig. S11).

Single-cell transcriptomic analysis revealed that CD163/LNP-FAPCAR intervention promoted M1 polarization via the Toll-like receptor activation and downstream NF-κB signaling, driving transcription of inflammatory mediators, including IL-6 (*SI Appendix*, Fig. S12). Notably, myeloid cell-specific IL-6 signaling has been implicated in the amelioration of NAFLD-associated fibrosis (33). This immune-metabolic reprogramming synergistically drives microenvironmental conversion toward a proresolution state. This study elucidated a mechanism by which αCD163/LNP-FAPCAR restored microenvironmental homeostasis in hepatic fibrosis by amplifying MMP12^+^ scar-resolving macrophage subpopulations. The functional dichotomy of SAM in liver disease remains contentious, with literature documenting both pathogenic and protective roles (34–37). Notably, Trem2 expression in SAM is indispensable for efficient clearance of apoptotic cells; hepatic fibrosis repair is impaired only upon concomitant depletion of SAM and SAM-like KC Trem2 (38). Our findings demonstrated that αCD163/LNP-FAPCAR treatment expanded the SAM compartment, with the LNP cohort showing enhanced MMP12 and additional SAM-associated molecule expression compared with untreated controls. Specifically, LNP administration enriched the reparative SAM subset (MMP12^+^MMP13^+^) (39, 40) while concomitantly reducing the profibrotic SAM population (CD9^+^) (34). Flow cytometric analysis validated the expansion of the CCR2^+^MMP12^+^MMP13^+^ population, which was accompanied by a contraction of MKI67^+^ proliferative and CD9^+^ scar-associated cells (Fig. 5*D*). Functionally, MMP12^+^ macrophage-conditioned medium markedly blunted fibrogenic gene expression in TGF-β1-stimulated HSCs (Fig. 5 *F*–*H*), underscoring the direct antifibrotic capacity of this specific subset. It is noteworthy that necrotic hepatocytes (necHC) specifically upregulate the “don’t eat me” ligand CD47. This feature distinguishes them from apoptotic Heps, thereby severely compromising macrophage-mediated clearance (41). We therefore posit a core mechanism of SAM dysfunction in fibrogenesis: CD47-mediated phagocytic inhibition impedes the induction of repair factors such as MMP12. The FAPCAR architecture employed in this study incorporated the CD28 costimulatory domain, which activated the PI3K pathway via phosphorylation of the p85 subunit, thereby enhancing integrin-mediated phagocytosis (*SI Appendix*, Fig. S1 *C*–*G*). This mechanism effectively circumvented CD47-dependent phagocytic resistance and drove SAM toward an MMP12-high reparative phenotype. Collectively, these findings furnish theoretical underpinnings for CAR-M-based antifibrotic therapeutics.

It can be inferred that a remarkable treatment process occurs during liver fibrosis, in which a large number of Heps undergo apoptosis and inflammatory factors (such as TGF-β1) are released (42), thereby activating HSCs. After the LNP treatment, FAPCAR-M is produced, which can target and eliminate activated HSCs. Furthermore, macrophages begin to clear apoptotic Heps (43) from the tissue and transform them into tissue repair cells. Finally, the changed tissue environment promotes the activation of Heps and the repair of fibrosis.

At the same time, we must carefully consider the potential immunogenicity and safety risks of this strategy. Exogenous antibody fragments may still pose a risk of inducing drug-resistant antibodies (ADA), which may affect targeting efficiency or trigger adverse events. In addition, off-target effects caused by the nonspecific distribution of LNP or low-level expression of FAP or CD163 on a very small number of other cell types still need to be ruled out. Further investigation is warranted to elucidate the extrahepatic expression patterns of αCD163/LNP-FAPCAR-transduced macrophages and their subsequent hepatic recruitment dynamics in the context of antifibrotic therapy. Furthermore, these LNPs therapy can increase the expression of MMP12 in macrophages, and the inducible effect of MMP12 depends on macrophage-mediated phagocytosis, although the exact mechanism remains unclear. Alternatively, the reduction in macrophage density following LNP treatment may result from the selective depletion of FAP-positive subpopulations; nevertheless, the underlying mechanisms necessitate further investigation. Research on those mechanisms will contribute to a deeper understanding of the role of macrophages in hepatic fibrosis and provide a foundation for novel therapeutic strategies.

**Fig. 6.**
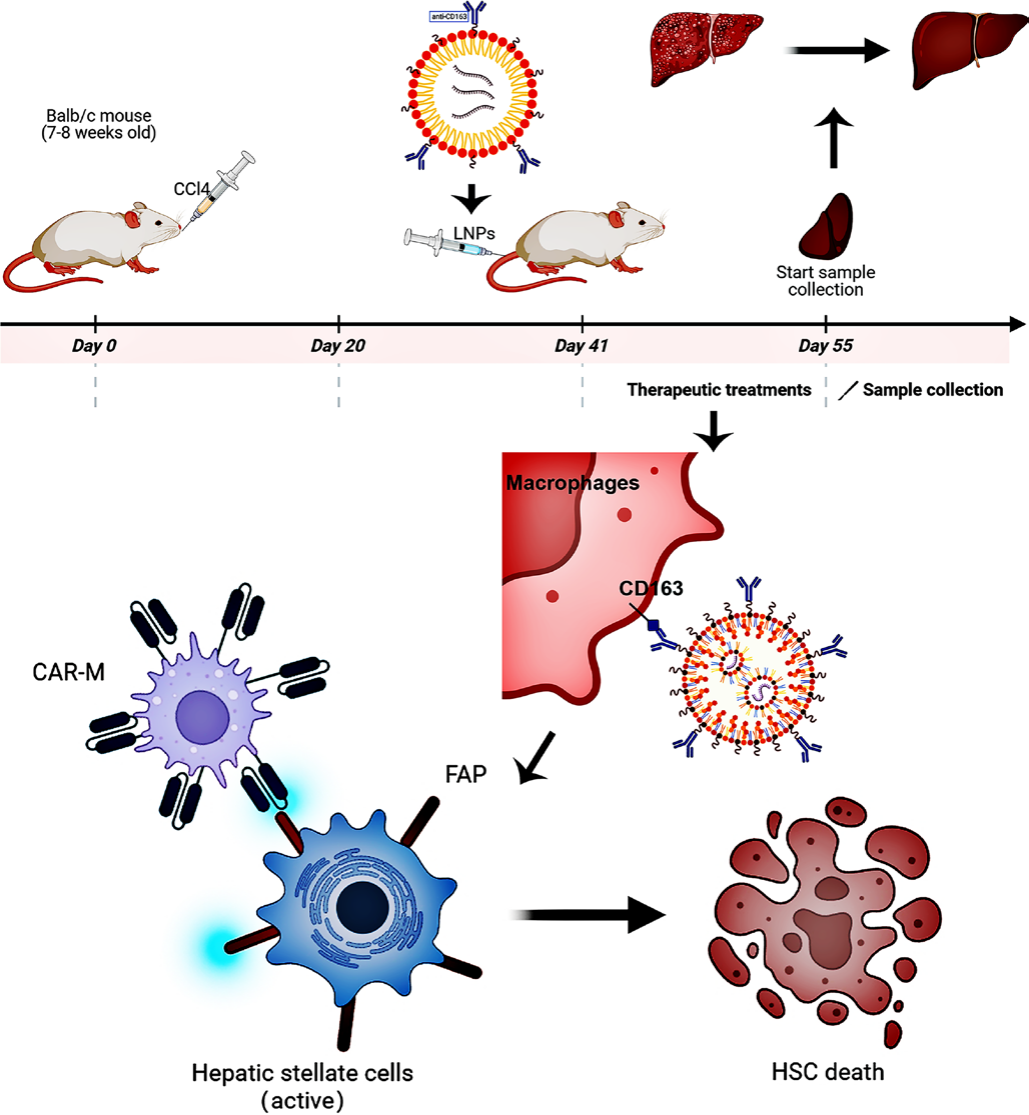
In situ engineering of CAR-macrophages for targeted depletion of FAP-expressing hepatic stellate cells in liver fibrosis.

In summary, although our study provides a promising strategy for macrophage-targeted mRNA CAR delivery in the treatment of liver fibrosis (Fig. 6), its clinical translation still requires a systematic evaluation of the mechanisms underlying long-term efficacy and potential safety issues. Future work will focus on optimizing dosing regimens to maintain efficacy, developing next-generation carriers with more complete human components to reduce immunogenicity, and conducting comprehensive preclinical safety evaluations to promote further development of this therapy.

## Materials and Methods

All materials and methods are described in detail in *SI Appendix*. The previously described cell culture, antibodies, lentiviral construction and infection, nanoliposome construction, determination of encapsulation efficiency, antibody coupling efficiency determination, assessment of cellular uptake, cell killing and phagocytosis, flow cytometry, bioluminescence, Immunohistochemistry (IHC), and Immunofluorescence (IF), real time PCR, western blot, animal research, blood chemistry analysis, pharmacokinetics analysis, the methods for single-cell sequencing, and data description are detailed in *SI Appendix*, *Supplemental Methods*.

### Ethics Approval and Consent to Participate.

The study was approved by the ethics committee of Shengjing Hospital, China Medical University, and conducted thereafter. All animal research was conducted in accordance with the Guiding Opinions on the Treatment of Laboratory Animals issued and the Laboratory Animal Guideline for Ethical Review of Animal Welfare issued by the National Standard GB/T35892-2018 of the People’s Republic of China, and all operations performed on mice have approved the China Medical University Standards for the Laboratory Animals Welfare and Ethical Review (Permit no: KT2022256). The collection of human samples was approved by the Hospital’s Ethics Committee (Permit no. 2024PS1753K)

### Consent for Publication.

All participants provided written informed consent before eligibility assessment, and they were informed that the data would be published.

## Supplementary Material

Appendix 01 (PDF)

## Data Availability

The data reported in this paper have been deposited in the OMIX, China National Center for Bioinformation/Beijing Institute of Genomics, Chinese Academy of Sciences (accession no: OMIX012254) (44).
