# Supplementary material for: mRNA-laden LNP-enabled in situ CAR-macrophage alleviates liver fibrosis via inhibiting activated HSCs and modulating the immune microenvironment
Source: Proc Natl Acad Sci U S A. 2026 May 29;123(22):e2534673123. doi: 10.1073/pnas.2534673123 (PMC13229182; doi:10.1073/pnas.2534673123)
Supplement: Supplementary file 1 — Appendix 01 (PDF) [file pnas.2534673123.sapp.pdf]

Supporting Information for

mRNA-Laden LNP-Enabled in situ CAR-macrophage alleviates liver fibrosis *via* inhibiting activated HSCs and modulating the immune microenvironment

Xin Huang[1#], Junfeng Hao[1,2#], Shuo Wang[3#], Botian Deng[2], Peng Wang[2], Qiuyu Zhao[4], Hongbo Liu[5*], Jiahe Wang[1*]

1 Department of Family Medicine, Shengjing Hospital of China Medical University, Shenyang, 110022, China;

2 Department of Nephrology, and Guangdong Provincial Key Laboratory of Autophagy and Major Chronic Non-communicable Diseases, Affiliated Hospital of Guangdong Medical University, Zhanjiang, 524001, China;

3 Department of Cardiology, Shengjing Hospital of China Medical University, Shenyang, 110022, China;

3 Department of Nephrology, Shengjing Hospital of China Medical University, Shenyang, 110022, China;

4 Key Laboratory of Ministry of Education for TCM Viscera-State Theory and Applications, Liaoning University of Traditional Chinese Medicine, Shenyang, 110847, China;

5 Third Department of Respiratory, Shengjing Hospital of China Medical University, Shenyang, 110022, China;

# These authors contributed to this work equally.

* Corresponding to:

Hongbo Liu (sysjlhb@163.com). The ORCID ID: https://orcid.org/0000-0002-3129-3710.

Jiahe Wang (wangjiahe_cmu@sina.com). The ORCID ID: https://orcid.org/0000-0001-6206-8404

This PDF file includes:

Supporting text
Figures S1 to S12
Tables S1 to S3
SI References

Other supporting materials for this manuscript include the following:

a modified nucleoside-inclusive mRNA encoding an FAP-targeted CAR sequence

Supporting text

SI Appendix, Supplemental Methods.

Patient Samples and Ethical Statement

The liver specimens were obtained from 15 liver fibrosis patients due to nonalcoholic fatty liver (NAFLD, n=4), hepatitis B (n = 3) or hepatitis C infection (n = 3), alcohol-associated liver disease (ALD, n=1), primary biliary cirrhosis (PBC, n = 2), and primary sclerosing cholangitis (PSC, n = 2) and from 15 respective neighboring non-cancerous of liver tissues (control group). All samples were collected at Shengjing Hospital of China Medical University between December 2024 and August 2025.

Cell Culture

The mouse HSC cell lines were procured from the YaJi Biological, the human HSC cell lines and THP-1 monocytes were procured from the Chinese Academy of Sciences' Cell Bank. They were cultured in DMEM supplemented with 10% fetal bovine serum (FBS) and 1% penicillin-streptomycin (P/S) solution. THP-1 monocytes (5 × 10^5 cells/mL) were seeded in complete RPMI-1640 with 80 nM phorbol 12-myristate 13-acetate (PMA) for 72 h to obtain adherent macrophages. JS-1 cells with puromycin resistance expressing luciferase and LX-2 cells expressing luciferase were incubated with puro to increase luciferase expression before use in the experiment. The cells were incubated at 37 °C in a 5% CO_2 humidified atmosphere.

Liver Fibrosis Models

Six to eight-week-old female Balb/c mice (Changsheng Bio, Liaoning) were maintained in SPF barrier rooms (22 ± 2 °C, 45-65 % humidity, 12 h light/12 h dark) with ad libitum access to standard chow and autoclaved water. To establish the CCL4 model, animals were randomized using a random-number table to receive twice-weekly i.p. injections of either vehicle (olive oil) or 10% CCL4 in olive oil (2 mL/kg) for 6 consecutive weeks. To induce bile duct ligation (BDL)-induced liver fibrosis, the mice were anesthetized with inhalation anesthesia using isoflurane. After disinfecting the skin, make a midline incision in the abdomen. Expose the common bile duct and ligate it with 5-0 non-absorbable sutures, with a duration of up to 4 weeks after surgery. To induce liver fibrosis through methionine deficiency and choline deficiency (MCD) diet, mice were fed either a normal diet or an MCD diet for a duration of up to 8 weeks. The mice received αCD163/LNP-FAPCAR treatment after the model was completed via an intravenous injection into the tail vein at a dose of 0.4 mg of mRNA per kg body weight, and the samples were collected after 14 days of treatment.

Primary cell culture

Bone-marrow myeloid progenitors were flushed from tibiae of C57BL/6 mice and matured into BMDMs using standard differentiation protocols with 5 ng/ml mM-CSF (HYP7085, MCE, China) to increase the purity of $F4/80^+$ macrophages, and continuously supplemented with cytokines to maintain vitality. Fresh medium was exchanged every 48-72 h, and fully differentiated BMDMs were collected on day 7. To obtain the primary Heps, we used the retrograde two-step collagenase perfusion method. In short, after intraperitoneal injection of avidin to anesthetize mice,

Hank's Balanced Salt Solution (HBSS) without $Ca^{2+}$and Mg^{2+} was perfused for 2 minutes. Then, HBSS containing 0.5 mM EGTA was perfused for an additional 10 minutes at 37 °C, followed by HBSS containing 5 mM $CaCl_2$ and 0.05% type IV collagenase (Sigma, C5138, USA) for 10 minutes. This infusion method was performed via the inferior vena cava and used the vascular system to deliver collagenase to most liver cells while maintaining their structural integrity. Following collagenase digestion, the liver was excised and gently homogenized with a syringe plunger in ice-cold DMEM. The filtrate was spun at 50 × g for 2 min; the pellet was retained, and Heps were distinguished from non-parenchymal cells based on their different cell densities. Then wash the precipitate with 20 mL of DMEM, suspend it in DMEM for cell counting, and continue culturing. Isolation of HSCs was performed by differential density-gradient centrifugation employing a Nycodenz medium (1.4×10^3 × g for 20 min at 4 °C), with concomitant cold-temperature fractionation of the non-parenchymal cell compartment to ensure optimal cell yield and functional preservation.

Lentiviral transduction

In short (1), lentiviral vectors were generated by cloning CAR constructs into a third-generation pTRPE backbone harboring a luciferase reporter and a puromycin resistance cassette via standard molecular cloning techniques. The CAR architecture comprises the CD8 leader sequence, (GGGGS) linker, CD8 hinge region, CD28 transmembrane and intramembrane domains, and the CD3ζ signaling domain, all under transcriptional control of the EF1α promoter. Viral particles were produced in HEK293T cells, with Vpx incorporated during packaging to enhance macrophage transduction efficiency. Following harvest, lentiviral supernatants were purified and concentrated. Unless otherwise indicated, target cell lines were transduced at a multiplicity of infection (MOI) of 3-5. Subsequently, the transduced cells were screened with puromycin, and cells with an expression rate exceeding 90% were selected for subsequent experiments.

RNA Synthesis and Lipid Nanoparticle Formulation

The FAP-CAR design incorporated a murine anti-FAP scFv (clone 73.3) linked to the cytoplasmic signaling domains of CD28 and CD3ζ (the sequence was in Supplementary Material 1). The sequence, codon-optimized for mammalian cells, was cloned into an IVT template plasmid encompassing a T7 promoter, 5' and 3' UTR elements, and a poly(A) tail. GeneChem (Shanghai, China) provided cloning and endotoxin-free plasmid preparation services. The mRNA production was facilitated using the MEGAScript T7 kit, substituting m1Ψ-5'-triphosphate for UTP, and incorporating a 101-nucleotide poly(A) tail. CleanCap® trinucleotide cap1 analog was incorporated cotranscriptionally during IVT to yield 5′-capped mRNA, which was purified using cellulose. The purified mRNA was analyzed on agarose gels and stored at -20°C. Cellulose-purified, m1Ψ-modified RNA was nano-encapsulated by microfluidic rapid-mixing with an ethanol-lipid cocktail (ionizable cationic lipid, helper phospholipid, cholesterol, and PEG-lipid) at acidic pH to form LNPs. The synthesis of the GFP/Luc/ZsGreen mRNA was the same as the FAP-CAR mRNA.

For ethanol-based lipid cocktail preparation, lipids were dissolved in absolute ethanol

at a molar ratio of 45:30:23.5:1.5 (ionizable lipid: DSPC: cholesterol: PEG-lipid). Notably, the elevated DSPC content (30 mol%) relative to conventional LNP formulations enhances interactions with monocyte-derived macrophages (MDMs), thereby facilitating targeting of circulating monocytes and promoting chemotaxis toward inflammatory sites (2).

The lipid mixture was subsequently combined with 6.25 mM sodium acetate buffer (pH 5.0) containing mRNA at an aqueous: organic phase ratio of 3:1 (v/v) using a microfluidic mixing device (Precision Nanosystems, Vancouver, BC). The resulting formulation was dialyzed against PBS (pH 7.4) for at least 18 hours, followed by concentration via ultrafiltration (Amicon centrifugal filter units; EMD Millipore, Billerica, MA). The final preparation was sterile-filtered through a 0.22 μm membrane and stored at 4°C until use. It is worth noting that the ionizable lipid employed in this study, heptadecan-9-yl 8-((2-hydroxyethyl)(8-(nonyloxy)-8-oxooctyl) amino) octanoate (molecular formula: $C_{44}H_{87}NO_5$; MW: 710.18), is an amino lipid architecture that substantially enhances endosomal escape efficiency (3). The antibody-targeted LNPs were prepared by conjugating LNP-mRNAs to purified rat anti-mouse CD163 and control using SATA-maleimide chemistry. Antibodies were stoichiometrically modified with SATA (N-succinimidyl S-acetylthioacetate) to introduce sulfhydryl groups, followed by post-insertion of DSPE-PEG-maleimide into pre-formed LNPs. This enabled site-specific thiol-maleimide bioconjugation, covalently tethering the sulfhydryl-activated antibody to the nanoparticle surface via stable thioether bond formation. After SATA deprotection with 0.5 M hydroxylamine, unreacted reagents were removed by passing the sample through a G-25 Sephadex desalting column. Subsequently, the antibody's active thiols were linked to the maleimide moiety via thioether coupling. Purification was achieved using an agarose CL-4B gel filtration column (Millipore Sigma). All ligand-decorated and control LNPs were kept at 4 °C and used without further processing. Size (z-average) and polydispersity index (PDI) were determined in triplicate by dynamic light scattering (Zetasizer Nano, Malvern) of particles diluted in 1 × PBS; values are listed in Supplementary Table 1. Zeta potential measurements were taken on the nanoparticle variants using a Zetasizer Nano. For TEM imaging, 5 μL of sample was adsorbed onto carbon-coated 400-mesh copper grids, blotted, air-dried, and negatively stained with 1 % aqueous uranyl acetate for 30 s. The average particle size was determined by measuring particle diameters in TEM images using a Hitachi TEM.

Synthesis of Ionizable Lipid

A solution of nonyl 8-((2-hydroxyethyl)amino)octanoate (0.150 g, 0.42 mmol), heptadecan-9-yl 8-bromooctanoate (0.217 g, 0.47 mmol), and N, N-diisopropylethylamine (0.061 g, 0.47 mmol) in ethanol (2 mL) was stirred at 65°C for 18 hours. Following cooling to ambient temperature, the solvent was removed under reduced pressure. The residue was partitioned between ethyl acetate and saturated aqueous $NaHCO_3$. The organic layer was separated, dried, and concentrated in vacuo. Purification by silica gel chromatography [0–100% gradient of 1% NH_4OH/20% MeOH in dichloromethane] afforded heptadecan-9-yl 8-((2-hydroxyethyl)(8-(nonyloxy)-8-oxooctyl)amino)octanoate (0.089 g, 30% yield)

as the desired product.

Determination of encapsulation efficiency (EE)

The mRNA encapsulation efficiency (EE) was quantified using the Quant-iT RiboGreen RNA assay kit (R32705, Thermo Fisher Scientific, China) according to the manufacturer's protocol. EE (%)=(fluorescence of total mRNA-fluorescence of free mRNA)/fluorescence of total mRNA × 100%.

Antibody coupling efficiency determination

The LNP of the conjugated antibody was prepared by the molar ratio of antibody to LNP 1:20, the total amount of antibody used was determined by the amount of LNP, the conjugated LNP was purified by the Sepharose CL-4B gel filter column, and the unconjugated free antibody was obtained, the total amount of free antibody was determined by BCA method, and the amount of antibody of the conjugated antibody LNP was detected by enzyme-linked immunosorbent assay (ELISA) method, combined with the standard curve for quantification. An efficiency table of antibody conjugation was available in Supplementary Table 2. Coupling rate formula :

$$\text{Conjugation rate}(\%)=\frac{\text{total antibody amount of conjugated LNP}}{\text{antibody amount-free antibody amount}}\times 100$$

***In Vitro* Transfection of Cells**

LNP-encapsulated GFP mRNA was prepared for *in vitro* transfection experiments. Log-phase BMDMs were plated at 1×10^4 cells per well in 96-well plates and transfected with 100 ng of the LNP-encapsulated GFP mRNA. The transfection efficiency was subsequently quantified by flow cytometry.

The preparation of LNP encapsulated His-tag-FAPCAR mRNA and controls was undertaken for *in vitro* transfection experiments. Log-phase BMDMs were seeded at 3 $\times 10^5$ cells per well in 6-well plates and then transfected with the respective preparations. The transfection efficiency was subsequently assessed by flow cytometry using a PE anti-His-tag antibody.

Assessment of Cellular Uptake

BMDM cells pre-stained with the live-cell dye expressing green fluorescent protein (GFP) were cultured at a density of 10^5 cells per well in 24-well plates and allowed to adhere overnight. Subsequently, the cells were incubated for four hours with Cy5-mRNA, LNP-Cy5-mRNA, and αCD163/LNP-Cy5-mRNA. After incubation, cells were washed three times with PBS and examined by confocal laser scanning microscopy.

***In Vitro* Killing Assay**

Before experiments, JS-1/LUC and LX-2/LUC cells were stimulated with 5 ng/ml TGF-β1 (Peprotech, USA) for 48 h, and the cells were screened for FAP^+ using a flow cytometer; then, the cells were co-cultured overnight with BMDMs at specified ratios. After a PBS rinse, cells were lysed and luciferase activity was measured on a PerkinElmer Victor X3 plate reader using the Promega E151A kit according to the manufacturer's instructions. A reduction in luciferase activity suggests that functional BMDMs successfully eliminated FAP^+ JS-1 cells. The killing efficiency was calculated using the formula: 100 – ((test RLU / average RLU of no T cell) ×100), where RLU denotes relative luminescent units.

In Vitro Phagocytosis Assay

The *in vitro* phagocytosis assay was conducted using pHrodo-labeled, opsonized FAP^+ JS-1 cells, and BMDMs were labeled with GFP (green fluorescence). Then the GFP^+BMDMs were treated with different LNPs (αCD163/LNP-Luc, LNP-FAPCAR, and αCD163/LNP-FAPCAR). FAP^+ JS-1 cells were washed twice with PBS and introduced to semi-adherent macrophages in a 12-well microtiter plate. The plates were then chilled for 15 minutes to facilitate cell contact. Subsequently, the plates were centrifuged at 150 g for 5 minutes at 4 °C. The supernatant was replaced with pre-warmed medium at 37°C, and the dishes were incubated at 37°C in a 5% CO_2 atmosphere to promote phagocytosis. The process was monitored using a live cell imaging system. Red fluorescence denoted specific phagocytosis of BMDMs on JS-1 cells labeled with pHrodo.

Flow cytometry

The cells were collected and dispensed into 1.5-mL Eppendorf (EP) tubes and incubated with antibodies. Following a PBS wash, cells were resuspended in 500 µL PBS + 1 % BSA and analyzed using a Becton Dickinson FACSCalibur flow cytometer (San Jose, CA, USA). The data were analyzed using FlowJo 10.4.2 software (BD Biosciences, San Jose, CA, USA). Flow Cytometry Gating Strategy: First, perform pre-processing to exclude debris/dead cells using FSC (cell size) and SSC (granularity). For homogeneous cell populations, set the negative gate using a negative control (unstained with the target antibody). For non-homogeneous cell populations, gating requires a pure positive region: use single control cells stained with the target antibody as a positive control.

Bioluminescence

In the liver fibrosis model, mice were intravenously administered with α CD163/LNP-Luc loaded with Luciferase or with α CD163/LNP-ZsGreen loaded with ZsGreen mRNA (via an intravenous injection of the tail vein at a dose of 0.4 mg of mRNA per kg body weight). Subsequently, the systemic Luciferase fluorescence signal was monitored using an IVIS imaging system at specific time points. The biodistribution of the injected particles was evaluated by imaging the entire organ using IVIS. Data was analyzed using Living Image software. To observe the expression of LNPs in the liver, we detected the ZsGreen after administering αCD163/LNP-ZsGreen loaded with ZsGreen mRNA at specific time points.

Blood Chemistry Analysis

A 200 µL blood sample was obtained from mice subjected to various treatments, and sera were prepared and stored at -80°C. Liver function was assessed by assaying hydroxyproline and alanine aminotransferase (ALT) levels. Additionally, the concentrations of multiple cytokines and chemokines in plasma were quantified using a multiplex protein array kit according to the manufacturer's protocols.

Biosafety evaluation _in vivo_

On the 15th day post-injection, blood was drawn for hematology and serum chemistry, and major organs were fixed, sectioned, and stained with H&E for histopathologic evaluation.

Histology, Immunohistochemistry (IHC), and Immunofluorescence (IF)

Liver specimens were fixed in 4 % neutral-buffered formaldehyde, paraffin-embedded, and cut into 4 μm sections for histology and IHC. Sirius red staining was applied post-deparaffinization using the corresponding kit. After rehydration, sections were stained for 1 h in Sirius red, rinsed six times with 0.5% acetic acid, dipped in absolute alcohol, dehydrated, and mounted. Standardized protocols were followed for the immunofluorescent staining of target proteins. Quantification of both IF and IHC staining was performed using ImageJ software in a blinded approach.

Reverse Transcription and Quantitative PCR (qPCR)

Total RNAs were isolated from cells or tissues using TRIzol reagent (Qiagen) according to the manufacturer's guidelines. cDNA was synthesized with TaqMan Reverse Transcription Reagents (Thermo Fisher). qPCR was run in triplicate on an ABI StepOnePlus 96-well system using TaqMan or SYBR Green Gene Expression assays (Thermo Fisher) as appropriate. Primer sequences were available in Supplementary Table 3.

Western blot

Cells were lysed in RIPA buffer (P0013B, Beyotime, China) on ice, vortexed, and clarified by centrifugation (15 000 × g, 4 °C, 25 min). Proteins were resolved on SDS-PAGE (100 V, 1.5 h) and transferred to PVDF membranes (Millipore, USA) at 50 V for 80 min. After 1 h blocking in 5 % non-fat milk, membranes were probed overnight at 4 °C with the indicated primary antibodies.

Measurement of Cytokine Production

Serum and culture supernatant cytokines were quantified using mouse- and human-specific ELISA kits. Samples were run in duplicate, standards on each plate, and absorbance was read at 450 nm.

Pharmacokinetics analysis

mRNA quantification was performed utilizing the QuantiGene 2.0 bDNA assay platform (Affymetrix). Blood-derived specimens were processed using the QuantiGene Sample Processing Kit before hybridization-based detection. A broad-range dilution series (1:100–1:1,250,000) was employed to validate linearity of quantification, with analytical replicates analyzed at each dilution point.

Single-Cell RNA Sequencing

Liver cells were dissociated and live cells were enriched using the Dead Cell Removal Kit (Miltenyi Biotec) for library preparation. Sequencing was conducted on the NextSeq 550 sequencer. The raw data were processed using the proprietary 10× Genomics CellRanger software (v4.0.0) with default parameters. The mkfastq command was initially used to demultiplex the raw BCL sequencing images using a provided sample sheet with the specified 10× barcodes. This process produced standard FASTQ files and metadata specific to 10× Genomics. Subsequently, CellRanger mapped the read data against the provided mouse reference genome (mouse reference dataset-gex-mm10-2020-A from 10× genomics). The scRNA-seq core cells were processed using the "Seurat" R package. Cells were disqualified if they contained genes detectable in fewer than 3 cells or were of low quality, with fewer than 200 detected genes. The gene expression of the core cells was normalized using a linear regression model, and a variance analysis was conducted to select the

top 2000 highly variant features. The "clustree" R package was used to cluster single-cell samples based on cell-specific gene characteristics. The Uniform Manifold Approximation and Projection (UMAP) algorithm was employed for dimensionality reduction analysis of the samples. Reference data-guided annotation, the CellMarker database, and previous studies were used to identify marker genes for different clusters manually. The "Seurat" R package was used for subsequent comparisons. Perform gene set enrichment analysis (GSEA) on differentially expressed genes (DEGs) between two groups using the R package "clusterProfiler ".

Data analysis

Statistical analyses were performed using GraphPad Prism 7.0 (GraphPad Inc., La Jolla, CA, USA). Continuous variables are presented as mean ± SD. One-way Inter-group differences were evaluated by one-way ANOVA. Tukey’s test was used for pairwise comparisons. ANOVA and the Mann-Whitney U test were used to compare data across groups. For all tests, a two-tailed P value of <0.05 was considered statistically significant.

Reagents and Tools Table

**Reagent/Resource**	**Reference or Source**	**Identifier or Catalog Number**
**Experimental Models**		
JS-1 cells (*m.musculus*)	YaJi Biological	YS2162C
LX-2 cells (*H. sapiens*)	Chinese Academy of Sciences' Cell Bank	SCSP-527
JS-1/LUC cells (*m.musculus*)	YaJi Biological	YS2595LUC
LX-2/LUC cells (*H. sapiens*)	YaJi Biological	YS110367C
THP-1 (*H. sapiens*)	Chinese Academy of Sciences' Cell Bank	SCSP-567
Balb/c mice	Changsheng Bio	N/A
**Antibodies**		
PE-conjugated anti-mouse CD80	Biolegend	60055
PE anti-mouse CD206	Biolegend	141706
APC anti-mouse F4/80	Biolegend	123116
APC anti-mouse CD45	Biolegend	147707
PE anti-mouse CD163	Biolegend	156704
PE anti-human CD68	Biolegend	333808
PE anti-mouse FAP	Bio-Techne	983802
anti-p-P50NF-κB	Affinity	AF3219
anti-P50NF-κB	Affinity	AF6217

anti-p-Syk	CST	44170
anti-Syk	CST	13198
anti-TGF-β1	CST	3711
anti-α-sma	CST	19245
anti-N-cadherin	CST	4061
anti-PCNA	CST	13110
anti-β-actin	Abcam	ab8226
anti-Collagen III	CST	94368
anti-MMP12	ThermoFisher	MA5-32011
anti-IL-6	ThermoFisher	AMC0864
anti-CD163	ThermoFisher	14-1631-82
anti-ALB	ThermoFisher	PA5-89332
anti-Desmin	ThermoFisher	MA5-32068
anti-CD45	ThermoFisher	MCD4530
anti-F4/80	ThermoFisher	14-4801-82
anti-SOX9	ThermoFisher	702016
anti-IL-10	ThermoFisher	ARC9102
anti-caspase3	ThermoFisher	700182
anti-FAP	ThermoFisher	PA5-99458
**Oligonucleotides and other sequence-based reagents**		
PCR primers	This study	Table3
**Chemicals, Enzymes, and other reagents**		
CCL4	MCE	HY-Y0298
MCD diet	Guanfeng Bio	A02082002BR
FBS	ThermoFisher	10270106
PMA	MCE	HY-18739
mM-CSF	MCE	HYP7085
type IV collagenase	Sigma	C5138
DMEM	ThermoFisher	41965062
RPMI-1640	ThermoFisher	
Quant-iT^{TM} RiboGreen RNA Assay kit	ThermoFisher	R32705
live cell dye	Yeasen	40721ES72

TGF-β1 protein	MCE	HY-P7117, HY-P7118
Hydroxyproline ELISA kit	Elabscience	E-BC-K062-M
ALT kit	Elabscience	E-BC-K235-M
RIPA lysis buffer	Beyotime	P0013B
IL-1β ELISA kit	Elabscience	E-EL-M0037/H0149
IL-6 ELISA kit	Elabscience	E-EL-M0044/H6156
IL-10 ELISA kit	Elabscience	E-EL-M0046/H6154
TGF-β1 ELISA kit	Elabscience	E-EL-UNEL-M0099/ UNEL-H0169
Cytochalasin D	MCE	HY-N6682
BAY 61-3606	MCE	HY-76474
Recombinant Mouse CD163 Protein Standard (His tag)	Abcam	ab322099
**Software**		
GraphPad Prism 7.0	https://www.graphpad.com	
CellRanger	https://www.r-project.org/	
Image J	https://imagej.nih.gov/ij/index.html	
FlowJo 10.4.2	https://www.flowjo.com	
**Other**		
Real-Time PCR System	ThermoFisher	
fluorescence microscope	Olympus Corporation	
IVIS Lumina III	PerkinElmer	
incucyte SX5	Sartorius	
Flow cytometer	Becton Dickinson	

Figures S1 to S12

Appendix Figure Legends

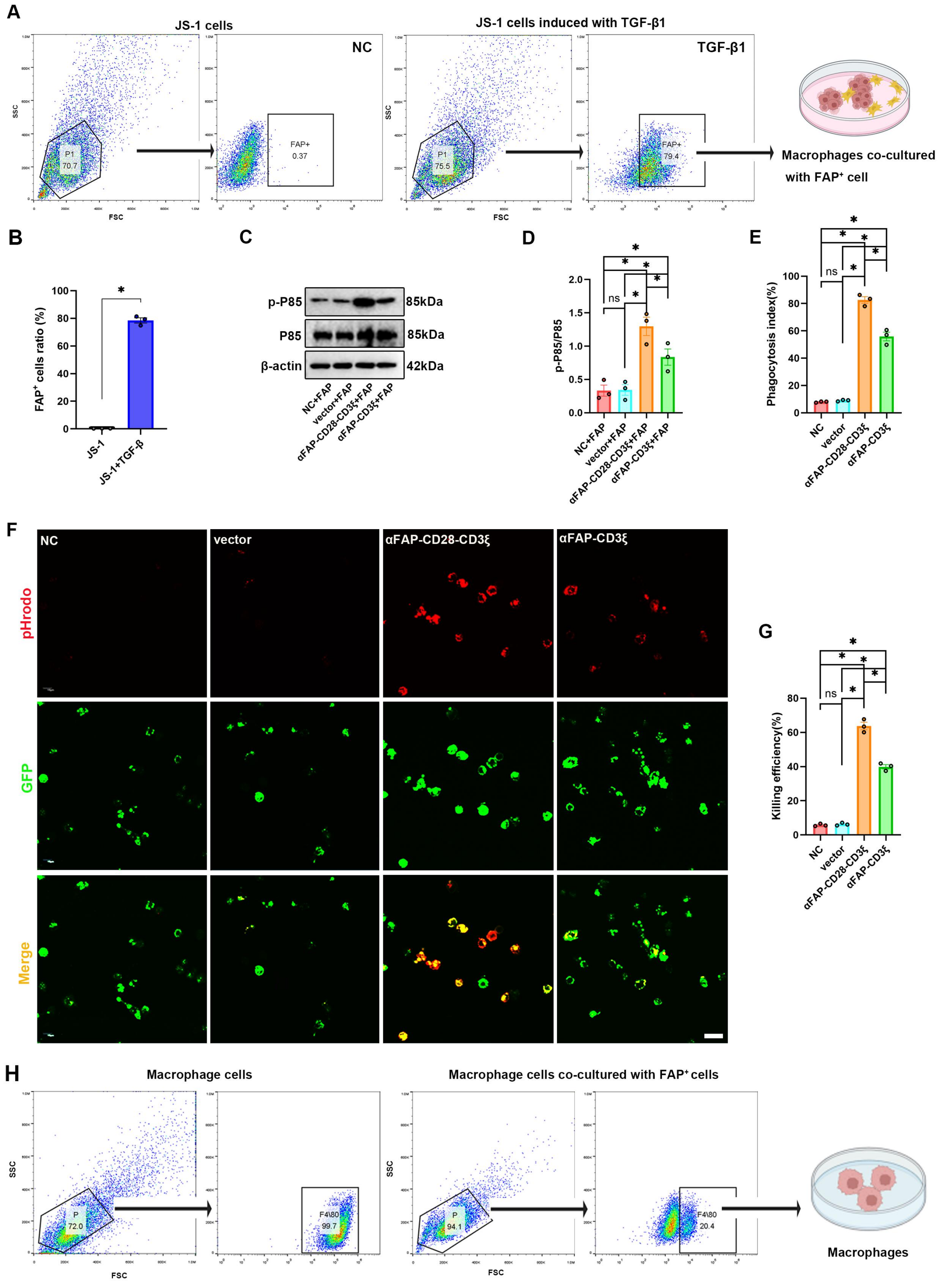

Appendix Fig 1. (A-B) Screened JS-1 cells with FAP$^+$ using a flow cytometer and the statistical analysis. JS-1 cells without antibody staining were used as negative controls. (C-D) The relative protein expression level of p-P85/P85 of BMDMs was analyzed using western blot. (E-F) Confocal microscopy images displayed the phagocytosis of FAP$^+$ JS-1 cells by BMDMs subjected to various formulations. (G) The cytotoxicity of BMDMs against target FAP$^+$JS-1 cells was assessed by co-culturing them overnight and calculating the killing

efficiency in biological replicates. (H) Screened BMDMs under the co-cultured system with FAP+ JS-1 cells using a flow cytometer. Gate strategy for flow cytometry: exclude debris/dead cells (left side) through FSC (cell size) and SSC (particle size). The left part represented the positive control of BMDMs, the right part represented the positive control gate to screen BMDMs in mixed cells. Data were representative of three independent experiments. Differences among groups were statistically evaluated using one-way ANOVA. Significance was indicated as *$P < 0.01$ and non-significance as ns $P > 0.05$.

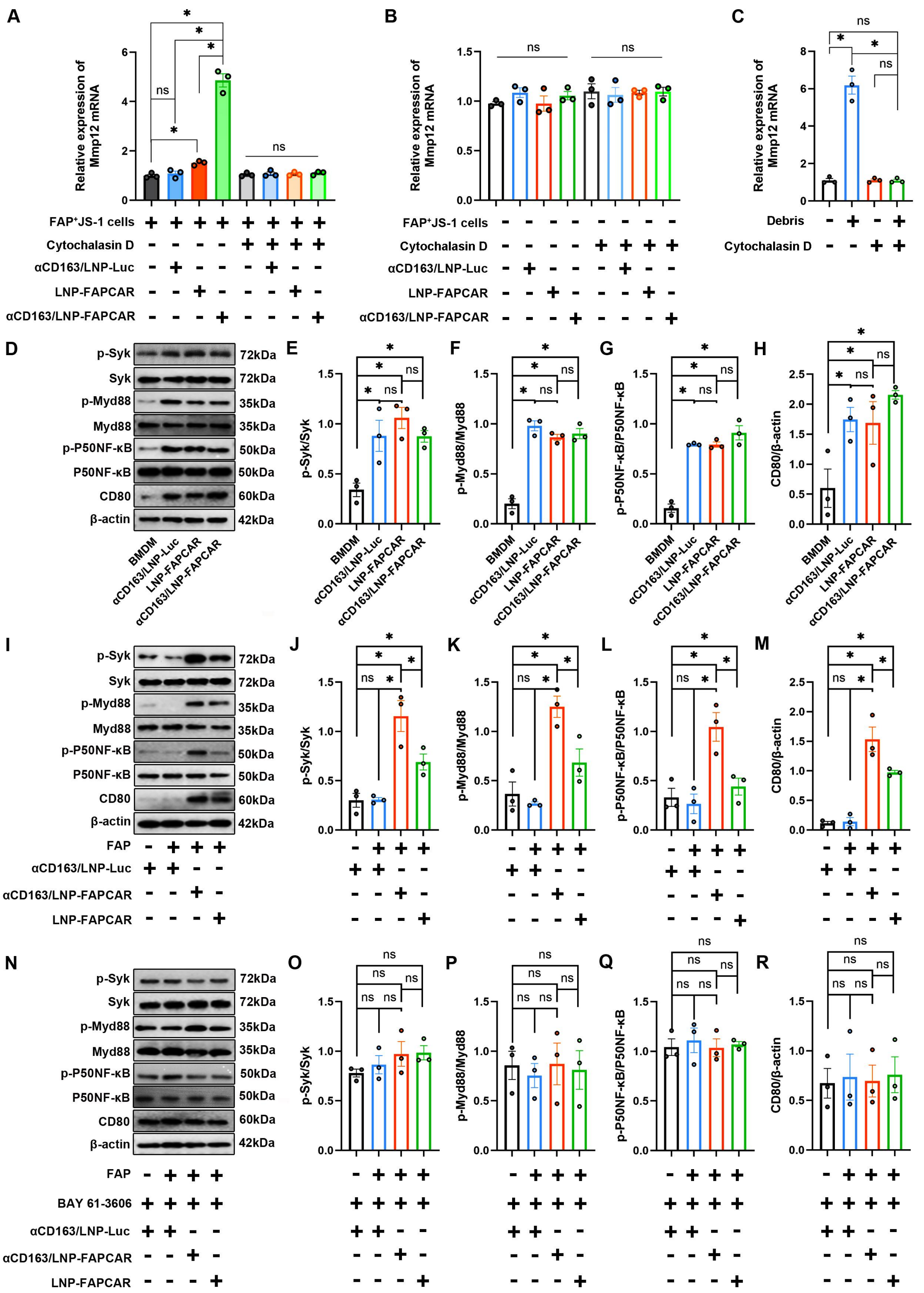

Appendix Fig 2. (A) The mRNA relative expression of *Mmp12* in screened BMDMs with Cytochalasin D (0.1 μg/mL for 24 h). (B) The mRNA relative expression of *Mmp12* in BMDMs alone with Cytochalasin D or not. (C) The mRNA relative expression of *Mmp12* in BMDMs with cell debris. (D-H) The relative protein expression level of p-Syk/Syk, p-Myd88/Myd88, p-P50NF-κB/P50NF-κB and CD80 of BMDMs alone was analyzed using western blot. (I-M) The relative protein expression level of p-Syk/Syk, p-Myd88/Myd88,

p-P50NF-κB/P50NF-κB and CD80 of BMDMs with FAP disposal and was analyzed using western blot. (N-R) The relative protein expression level of p-Syk/Syk, p-Myd88/Myd88, p-P50NF-κB/P50NF-κB and CD80 of BMDMs with FAP disposal and Syk inhibitor (BAY 61-3606, 2 μM for 24 h) was analyzed using western blot. Data were representative of three independent experiments. Differences among groups were statistically evaluated using one-way ANOVA. Significance was indicated as *P < 0.01 and non-significance as ns P > 0.05.

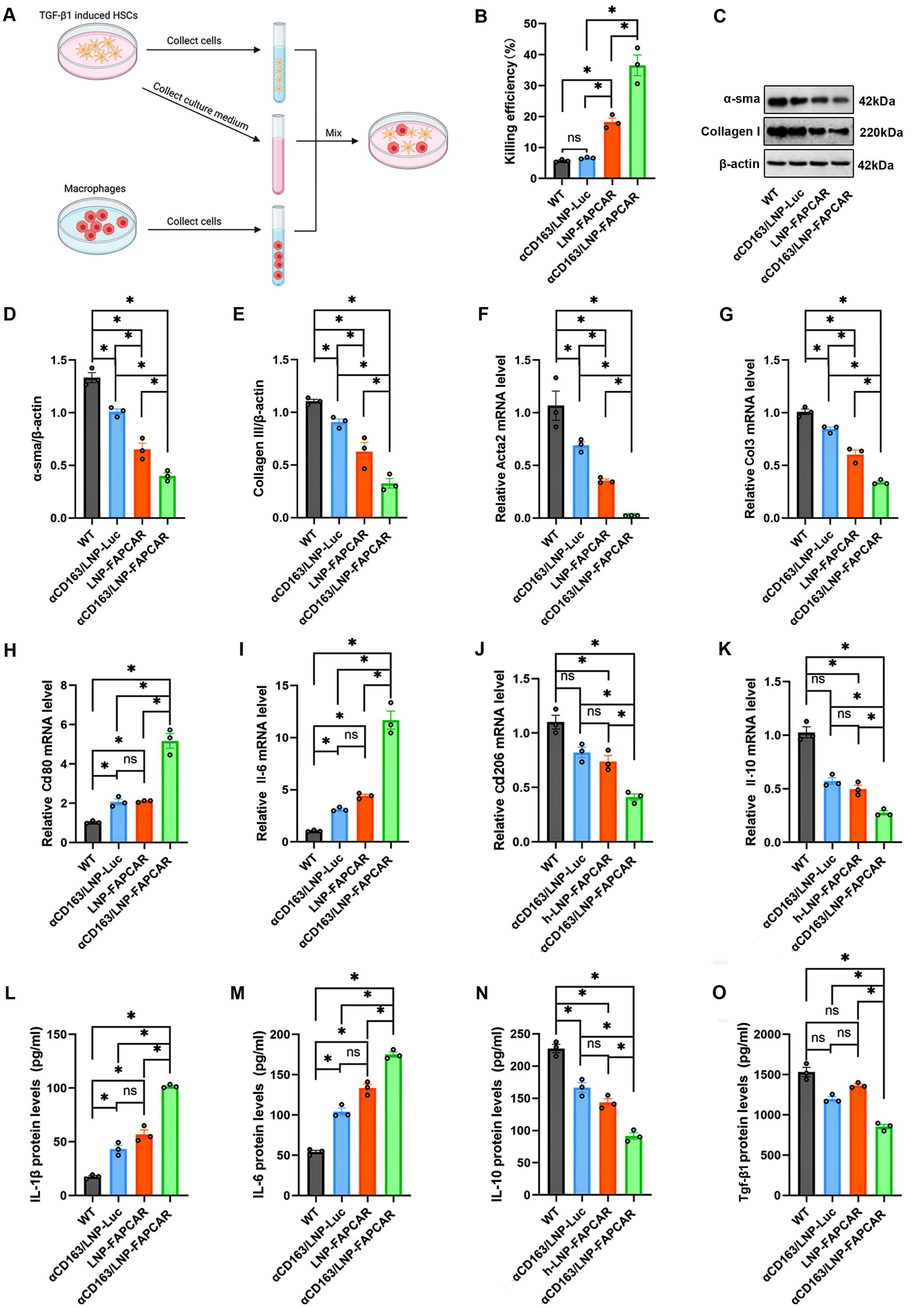

Appendix Fig 3. (A) The co-culture mode diagram of JS-1 cells and BMDM cells under the simulated fibrosis state induced with TGF-β1 *in vitro*. (B) The cytotoxicity of BMDMs against target JS-1 cells was assessed by co-culturing them overnight and calculating the killing efficiency in biological replicates. (C-E) The relative protein expression level of α-sma and Collagen-III of survived JS-1 cells was analyzed using western blot. (F-G) The qPCR analysis of *Acta2* and *Col3* expression in the survived JS-1 cells. (H-K) The qPCR analysis of

Cd80, *Il-6*, *Cd206*, and *Il-10* expression in the screened BMDMs. (L-O) The protein levels of IL-1β, IL-6, IL-10, and TGF-β1 of the supernatant of the screened BMDMs were measured through ELISA. Data were representative of three independent experiments. Differences among groups were statistically evaluated using one-way ANOVA. Significance was indicated as **P* < 0.01 and non-significance as ns *P* > 0.05.

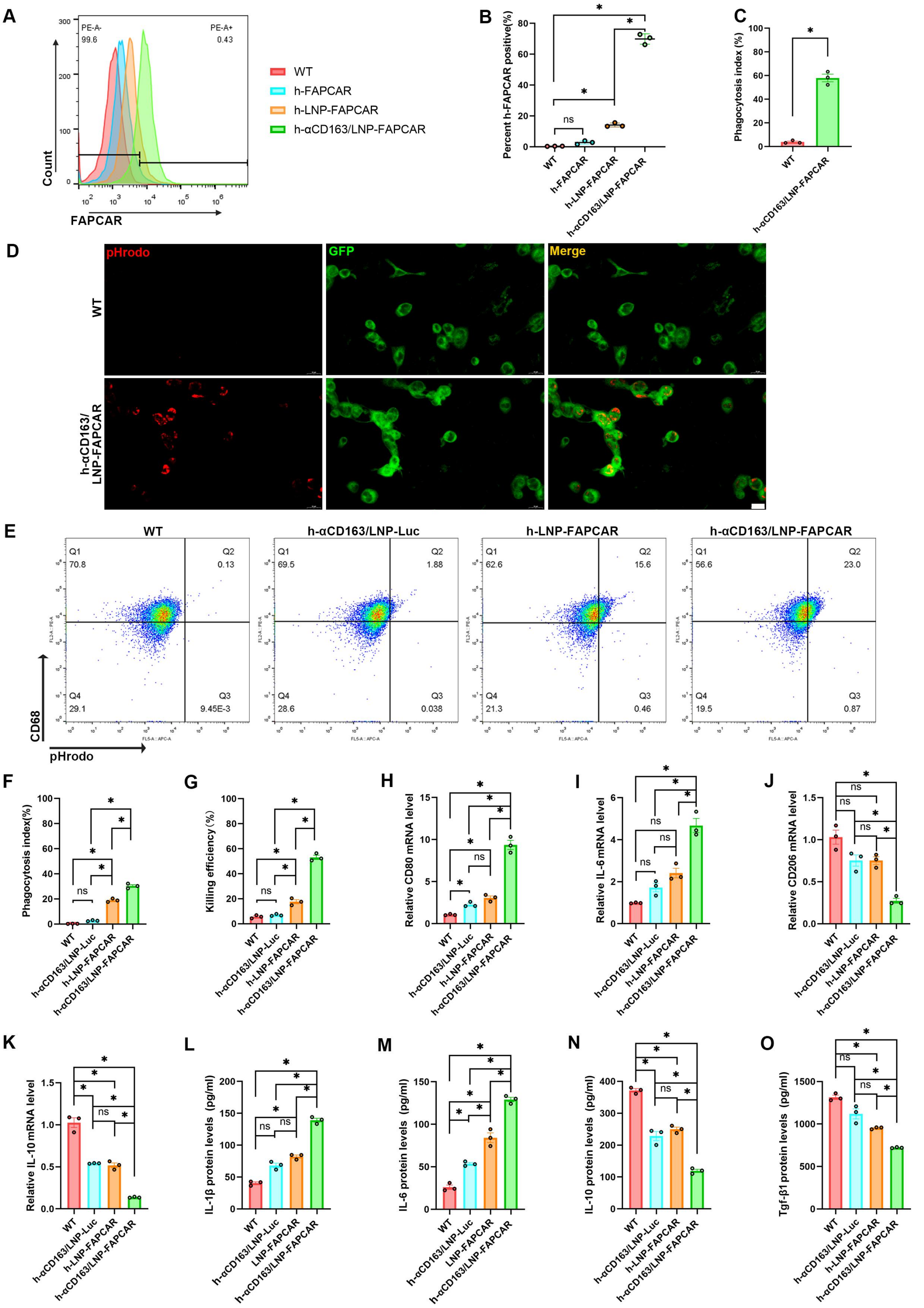

Appendix Fig 4. (A-B) Quantification of FAPCAR positive staining in macrophages (THP-1

cells induced with PMA for 24 h) with different treatments and the analysis across biological replicates. (C-D) Confocal microscopy images display the phagocytosis of FAP^+ LX-2 cells by macrophages subjected to various formulations and the quantitative analysis of the phagocytic index (scale bar=10 μm). (E-F) Flow cytometry was employed to detect the phagocytic activity of macrophages in response to various formulations, using pHrodo-labeled FAP^+ LX-2 cells and statistical analysis. (G) The cytotoxicity of macrophages against target FAP^+ LX-2 cells was assessed by co-culturing them overnight and calculating the killing efficiency in biological replicates. (H-K) The qPCR analysis of *CD80*, *IL-6*, *CD206*, and *IL-10* expression of the screened macrophages. (L-O) The protein levels of IL-1β, IL-6, IL-10, and TGF-β1 of the supernatant of the screened macrophages were measured through ELISA. Data were representative of three independent experiments. Differences among groups were statistically evaluated using one-way ANOVA. Significance was indicated as $*P < 0.01$ and non-significance as ns $P > 0.05$.

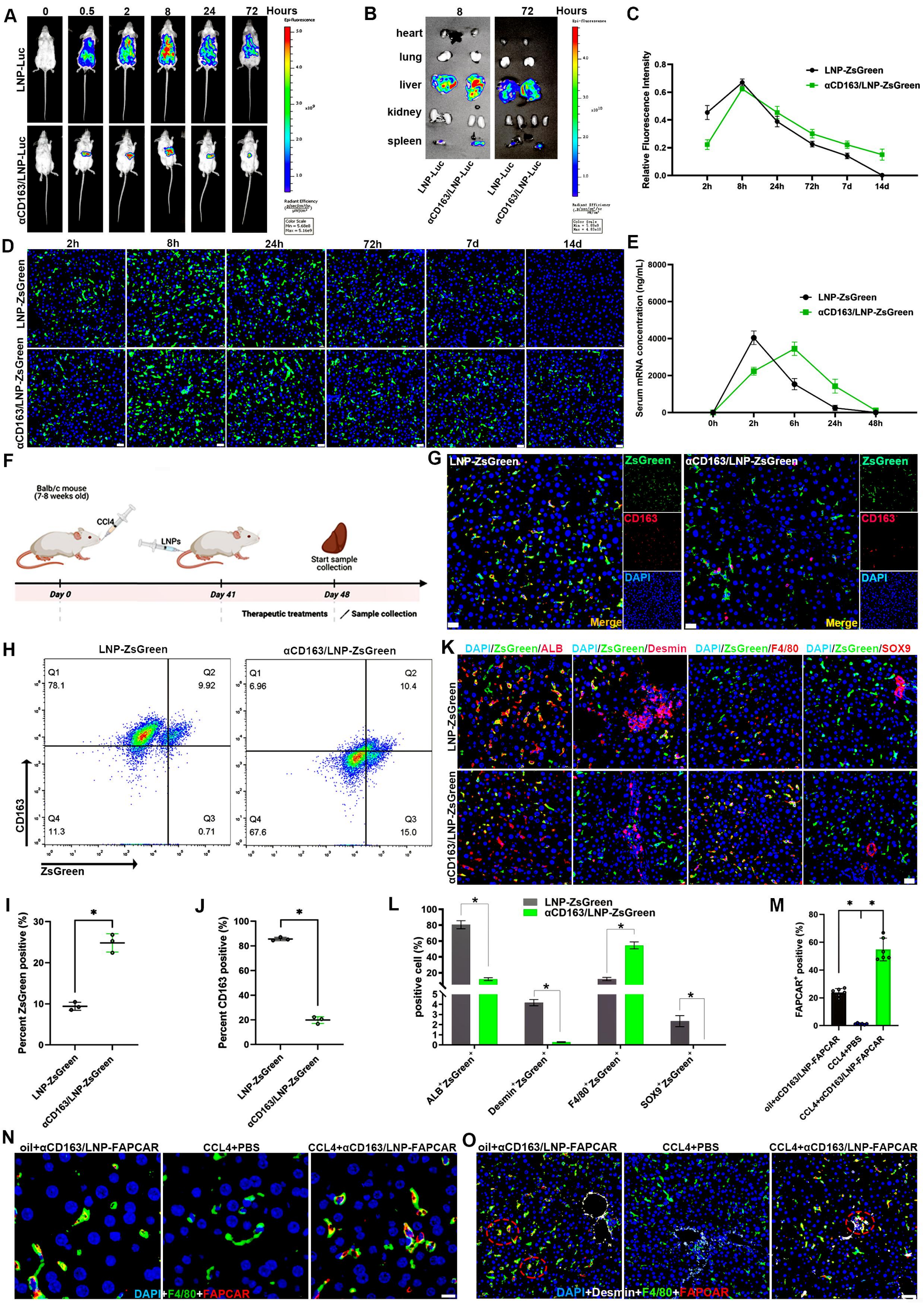

Appendix Fig 5. (A) Bioluminescence analysis of fibrotic mice induced by CCL4 at 0, 0.5, 2, 8, 24, and 72 hours post-intravenous injection of 40 μg of luciferase mRNA encapsulated in LNP (LNP-Luc and αCD163/LNP-Luc). (B) The luciferase activity of major organs at 8 and 72 hours. (C-D) IF images depicting ZsGreen expression in CCL4-induced fibrotic mice post-intravenous injection of 40 μg of ZsGreen mRNA encapsulated in LNP-ZsGreen and αCD163/LNP-ZsGreen groups at different times, and the statistical analysis. (E) The mRNA levels in serum over time. (F)

A schematic representation of *in vivo* delivery experiments. (G) IF co-localization images demonstrating the localization of CD163 (red) and ZsGreen in the fibrotic mouse liver (scale bar=20 μm). (H-J) Flow cytometry analysis of ZsGreen and CD163 expression in the screened macrophages and the analysis. (K-L) IF images depicting the co-staining with markers for liver cells (ALB^{+}), HSCs (Desmin^{+}), macrophages (F4/80^{+}), bile duct cells (SOX9^{+}), and the statistical analysis. (scale bar=20 μm). (n=3). (M-N) IF staining of FAPCAR (red) in liver tissues, highlighting F4/80^{+} (green) macrophages (scale bar=10 μm) and the statistical analysis. (n=6). (O) The triple IF staining of liver tissue sections using Desmin^{+} (white), F4/80^{+} (green), and FAPCAR^{+} (red) (scale bar=20 μm). (n=3). Differences among groups were statistically evaluated using one-way ANOVA. Significance was indicated as *$P < 0.01$ and non-significance as ns $P > 0.05$.

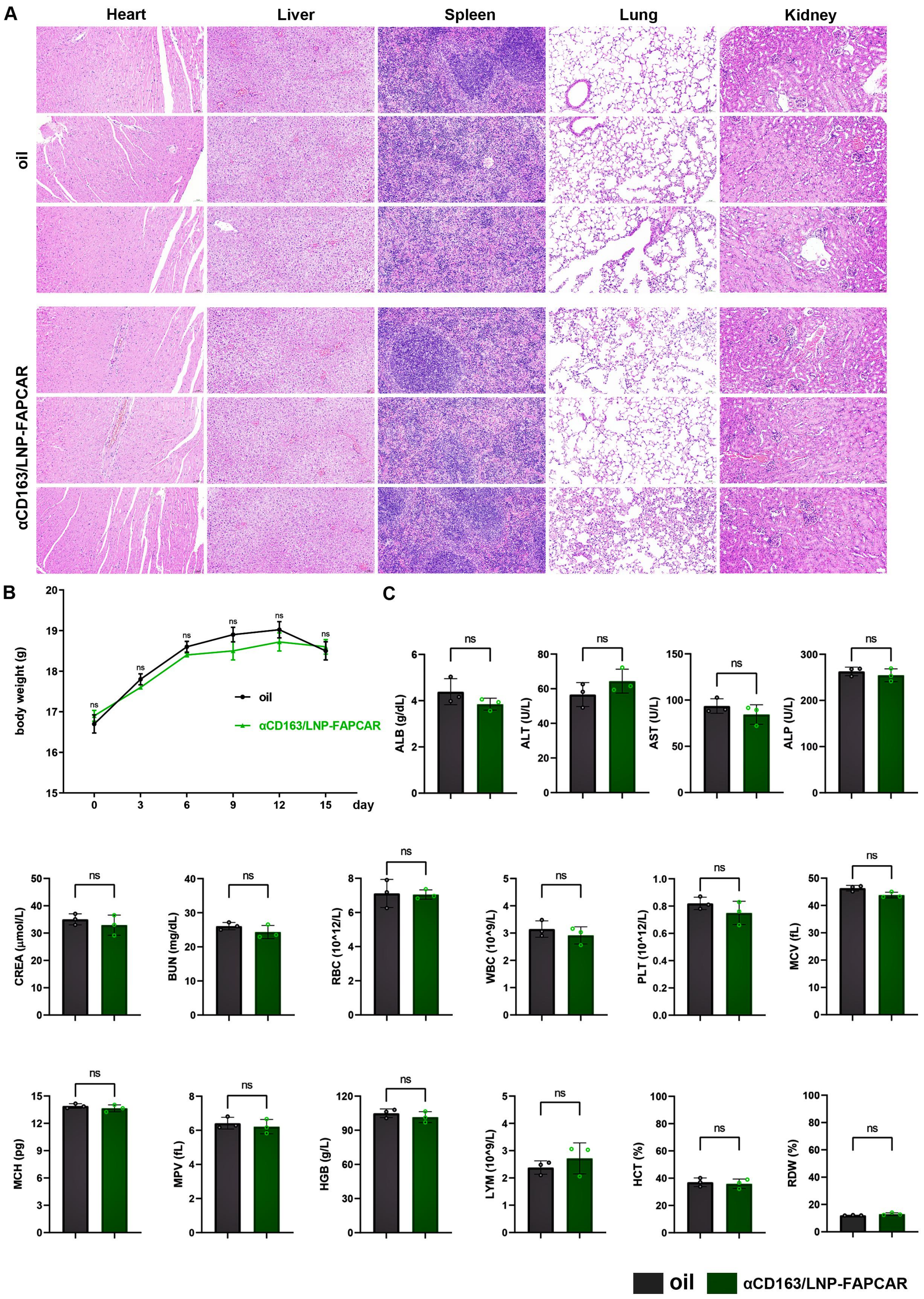

Appendix Fig 6. (A) H&E staining was performed to evaluate pathological changes in the heart, liver, spleen, lungs, and kidneys of the mice treated with oil and αCD163/LNP-FAPCAR (scale bar=20 μm). (B) The weight of the mice in different groups. (C) Routine blood test and biochemical evaluation of the mice after different treatments. Data were representative of three independent experiments. Differences among groups were statistically evaluated using one-way ANOVA. Significance was indicated as *P < 0.01 and

non-significance as ns $P > 0.05$.

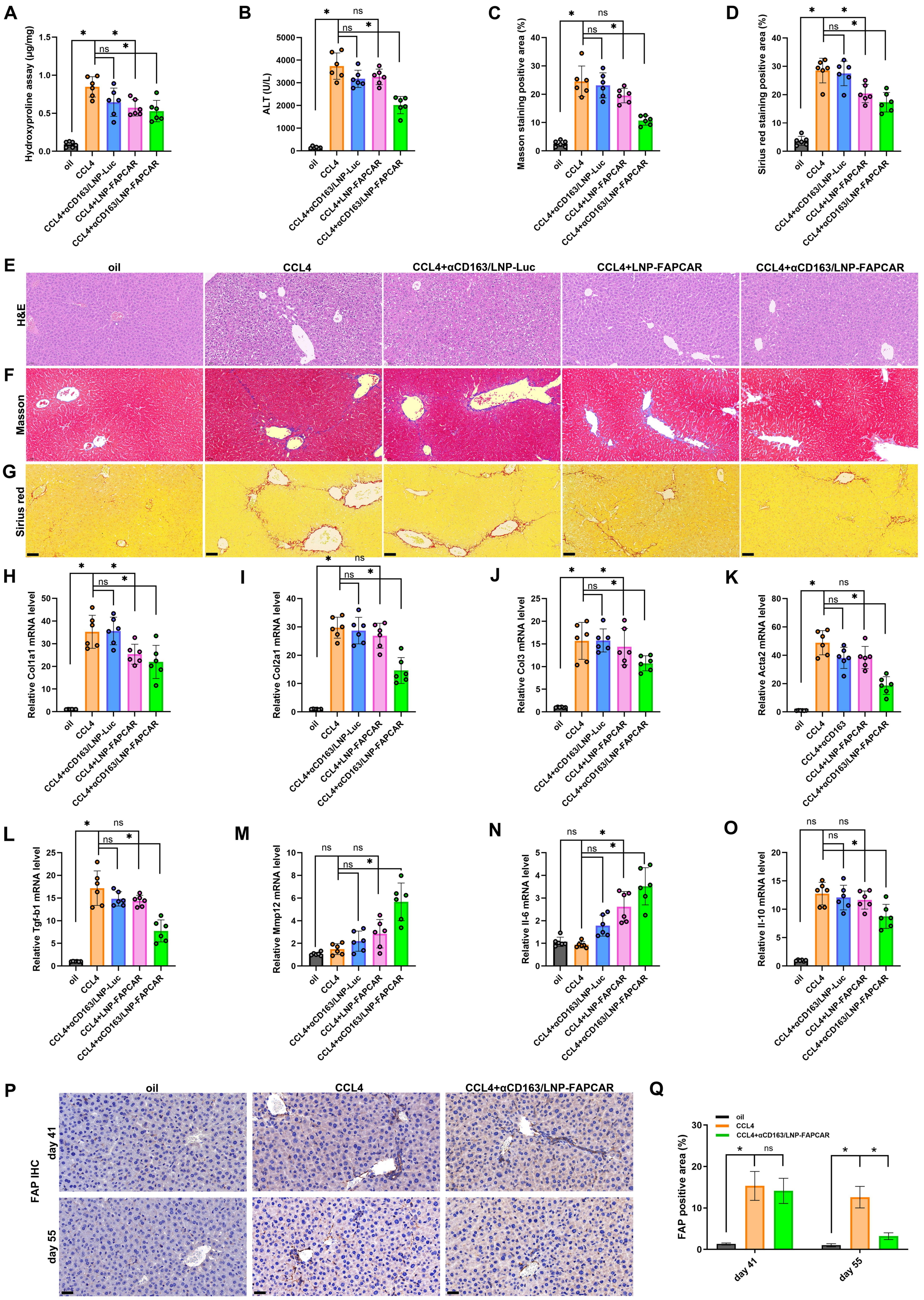

Appendix Fig 7. (A) Hydroxyproline quantification of the mice. (B) The serum ALT level determination. (C-G) H&E, Masson's trichrome, and Sirius red staining (scale bar=50 μm) with corresponding quantitative images. (H-O) The qPCR analysis of *Col1a1*, *Col2a1*, *Col3, Acta2, Tgf-b1, Mmp12, Il-6* and *Il-10* in liver tissues derived from the mice. (P-Q) The IHC staining of FAP (scale bar=50 μm) with corresponding quantitative images at different time.

Data were representative of six independent experiments. Differences among groups were statistically evaluated using one-way ANOVA. Significance was indicated as $*P < 0.01$ and non-significance as ns $P > 0.05$.

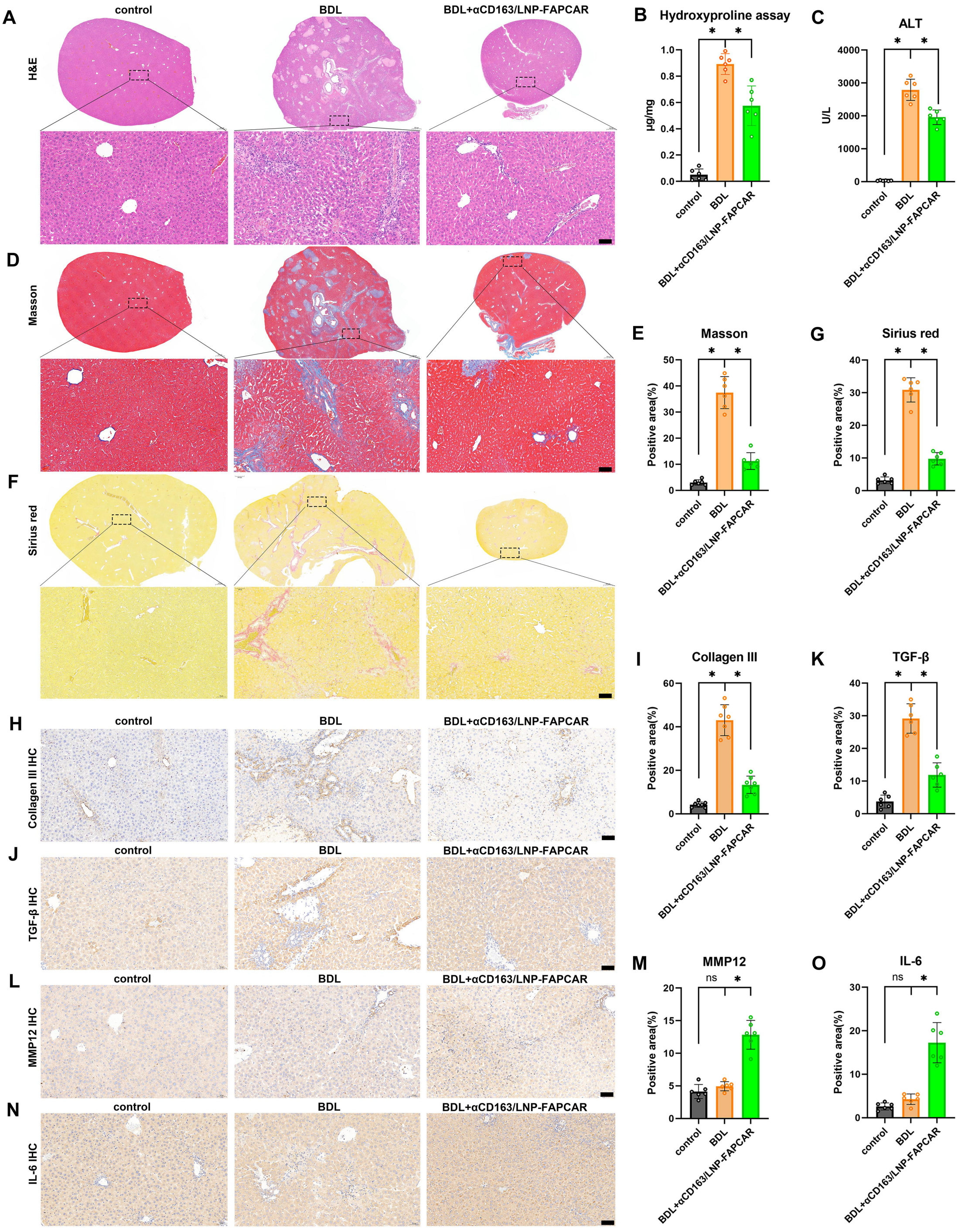

Appendix Fig 8. (A) H&E staining of the liver of the BDL model. (B) Hydroxyproline quantification of the mice. (C) The serum ALT level determination. (D-G) Masson's trichrome and Sirius red staining with corresponding quantitative images. (H-O) The IHC staining of Collagen III, TGF-β1, MMP12, and IL-6 of the liver of the mice with corresponding quantitative images. (scale bar=50 μm). Data were representative of six independent

experiments. Differences among groups were statistically evaluated using one-way ANOVA. Significance was indicated as *$P < 0.01$ and non-significance as ns $P > 0.05$.

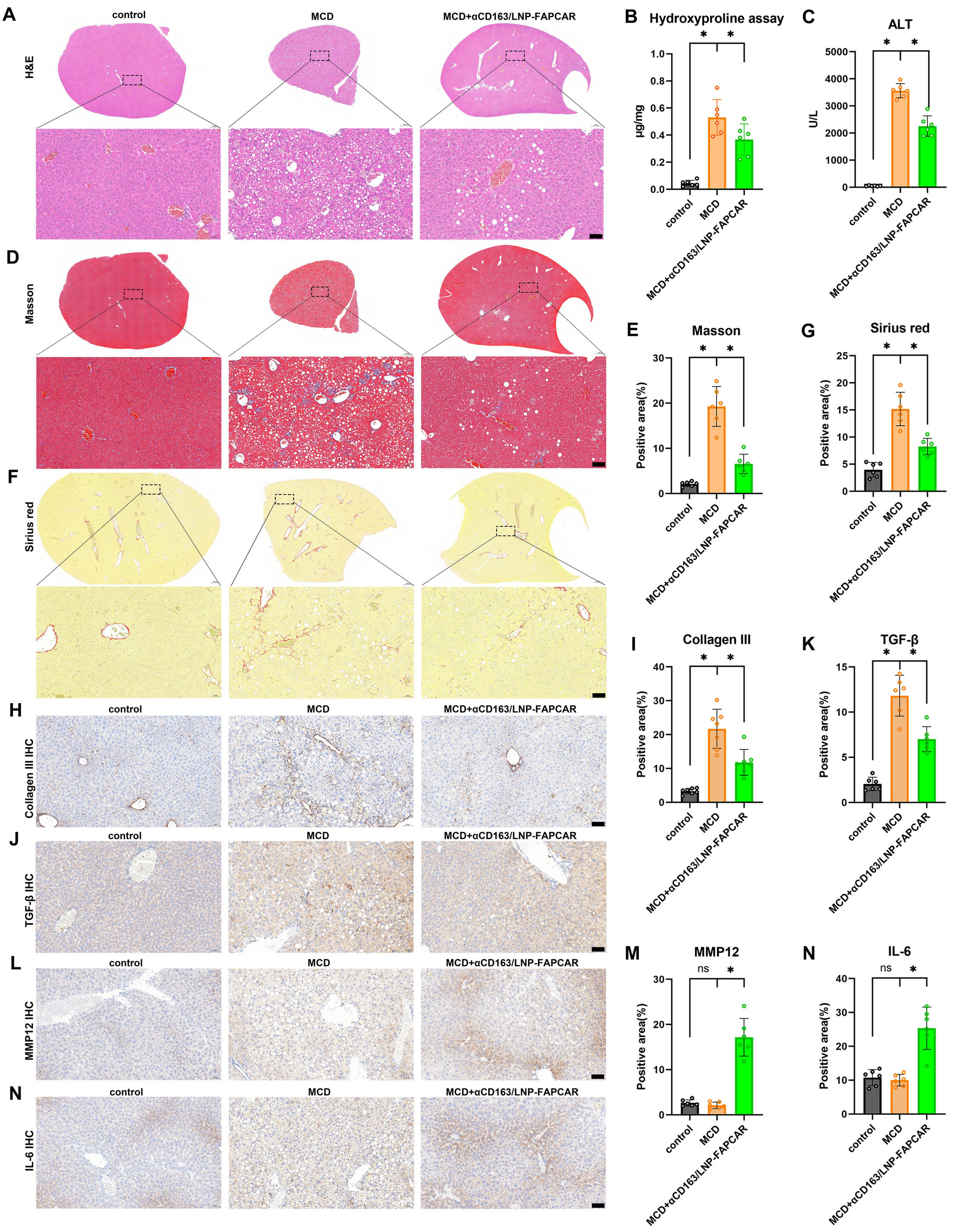

Appendix Fig 9. (A) H&E staining of the liver of the MCD model. (B) Hydroxyproline quantification of the mice. (C) The serum ALT level determination. (D-G) Masson's trichrome and Sirius red staining with corresponding quantitative images. (H-O) The IHC staining of Collagen III, TGF-β1, MMP12, and IL-6 of the liver of the mice with corresponding quantitative images. (scale bar=50 μm). Data were representative of six independent experiments. Differences among groups were statistically evaluated using one-way ANOVA. Significance was indicated as *$P < 0.01$ and non-significance as ns $P > 0.05$.

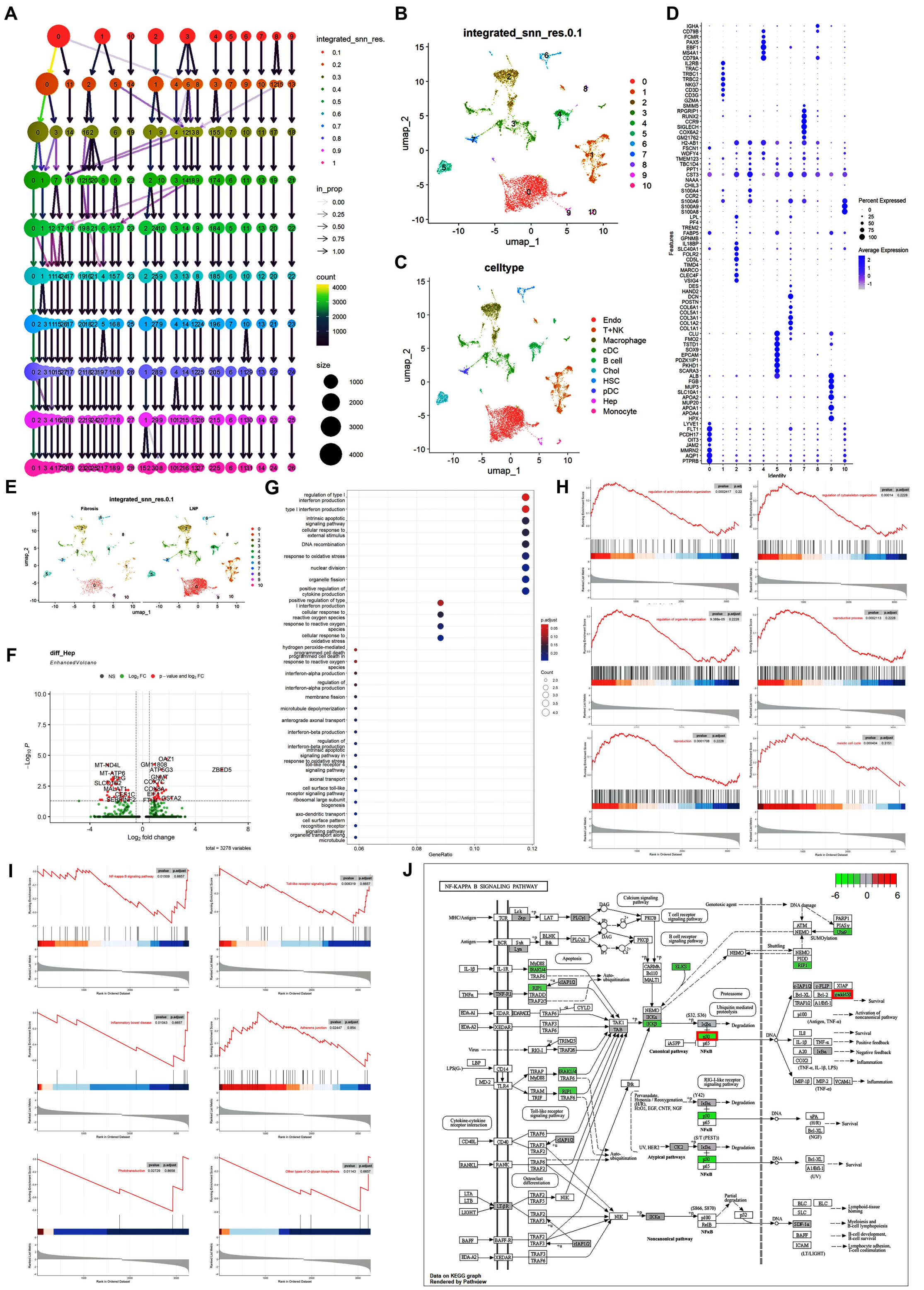

Appendix Fig 10. (A) The liver tissue cells were isolated with a selected dimensional reduction coefficient of 0.1. (B-E) The method identified 11 unique cell clusters (cluster 0-10). (F) The transcriptomic differences of Heps between Fibrosis and LNP groups. (G) The GO enrichment analysis on the DEGs of Heps. (H) The GSEA analysis of GO terms of Heps. (I) The GSEA analysis of the KEGG of the DEGs of Heps. (J) The p50 and gadd45b involved in the NF-κB pathways.

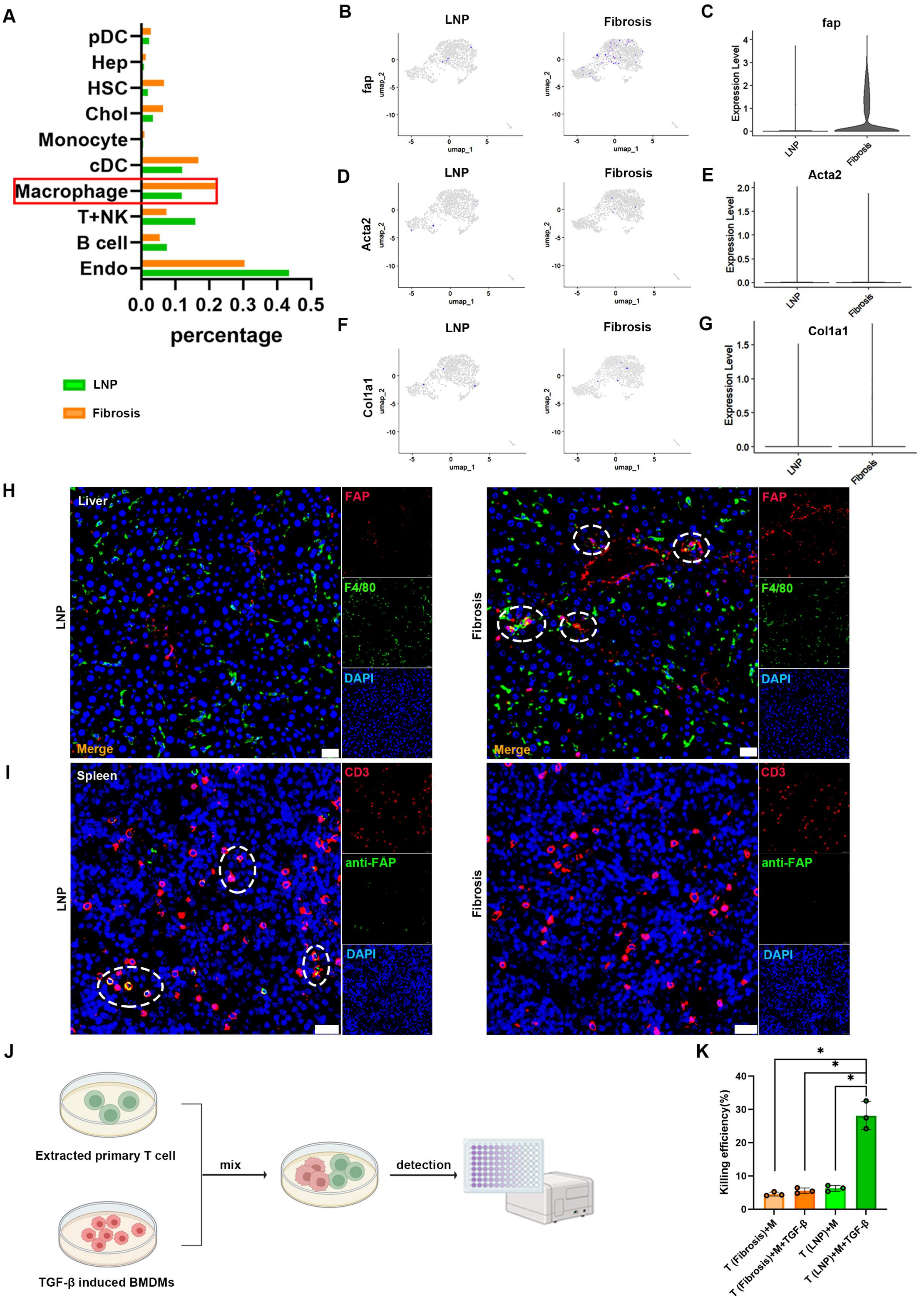

Appendix Fig 11. (A) Comparative analysis of cell proportions across groups. (B-G) Feature plot of the *Fap*, *Acta2,* and *Col1a1* gene expression in the macrophage subgroups and the statistical analysis. (H) The fluorescence confocal microscopy of FAP^+ (red), $F4/80^+$ (green), and DAPI of the macrophages in the liver tissues (scale bar=20 μm). (I) The fluorescence confocal microscopy of $CD3^+$ (red), anti-FAP^+ (green), and DAPI of the T cells in the spleen tissues (scale bar=20 μm). (J) The co-culture system of the extracted primary T cells and

TGF-β1-induced BMDMs. (K) The cytotoxicity of T cells against TGF-β1-induced BMDMs was assessed by co-culturing T cells overnight and calculating killing efficiency in biological replicates. The data were representative of three independent experiments. Differences among groups were statistically evaluated using one-way ANOVA. Significance was indicated as *P < 0.01 and non-significance as ns P > 0.05.

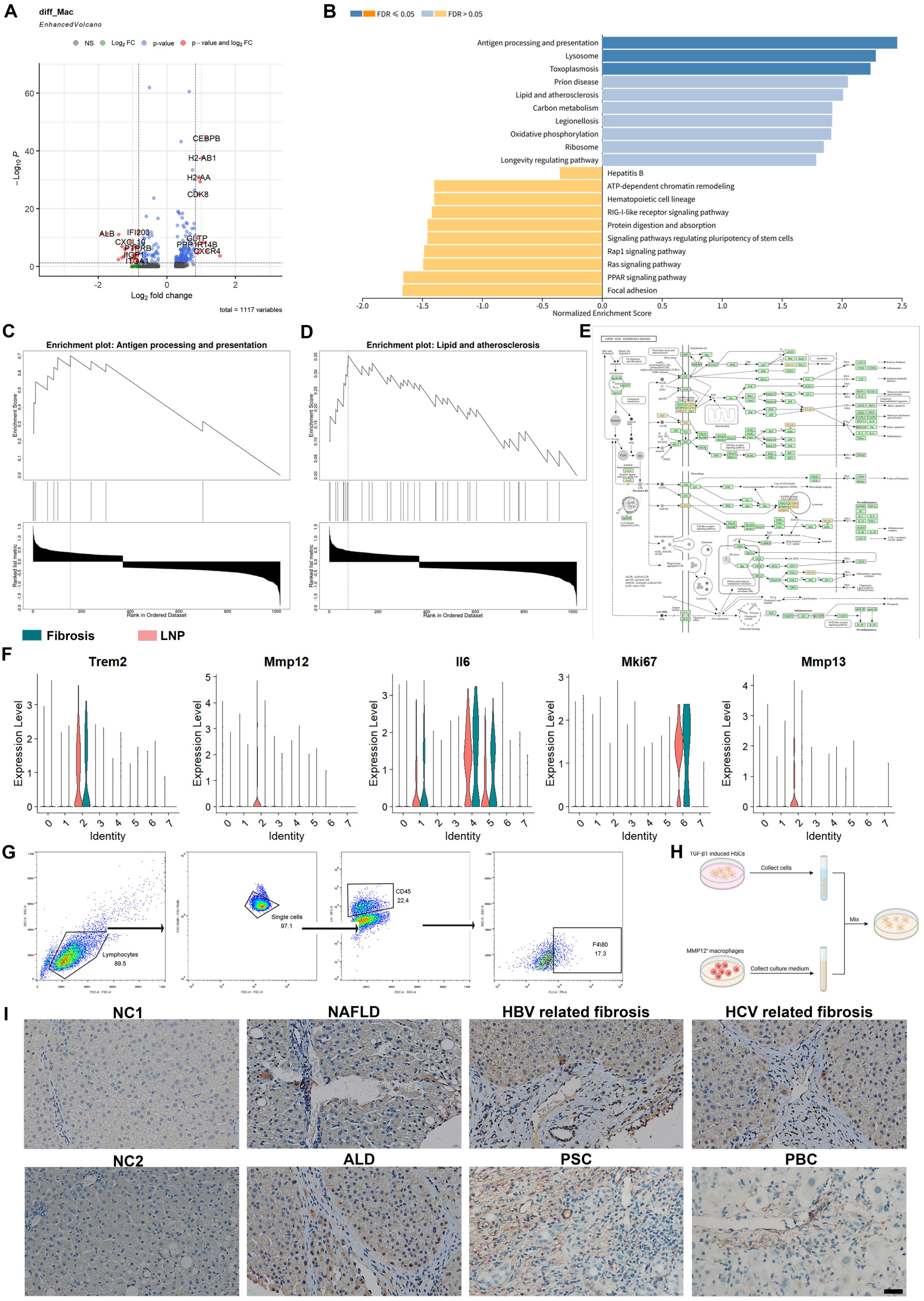

Appendix Fig 12. (A) The transcriptomic differences of macrophages between Fibrosis and LNP groups. (B) The GSEA enrichment of the DEGs of macrophages. (C) The Antigen processing and presentation enrichment plot. (D) The Lipid and atherosclerosis enrichment plot. (E) The Lipid and atherosclerosis enrichment pathways. (F) Vlnplot analysis of the markers gene expression in various macrophage clusters between the Fibrosis (blue) and LNP (pink) groups. (G) F4/80$^+$ macrophages from the mouse liver through flow cytometry screening. (H) The co-culture system of the macrophages and TGF-β1 induced HSCs. (I) liver fibrosis tissues from patients with different etiologies and performed IHC staining of FAP.

Tables S1 to S3

Appendix Table 1. Parameter of the diameter (z-average) and PDI of each LNP formulation (± standard deviation)

Sample	Diameter (nm)	PDI
LNP	117.01±2.78	0.15±0.026
αCD163/LNP	132.05±3.10	0.176±0.017

Appendix Table 2. Efficiency table of antibody conjugation in different batches

Batch	Total Lipid Concentration in LNPs (mg/ml)	Volume of LNPs (ml)	Amount of CD163 Antibody for LNP Conjugation (mg)	Free Antibody Amount (mg)	Amount of Antibody Conjugated to LNPs (mg)	Conjugation Rate (%)
1	4.836	2	0.312	0.182	0.119	91.54
2	4.077	2	0.264	0.153	0.103	92.79
3	4.358	2	0.278	0.154	0.114	91.94

Appendix Table 3. Primer sequences used in real-time PCR analysis

**Target gene**	**Forward primer (5'–3')**	**Reverse primer (5'–3')**
mus-Cd80	TGCTGCTGATTCGTCTTTCAC	GAGGAGAGTTGTAACGGCAAG
mus-Il-1β	AGAGCTTCAGGCAGGCAGTA	AGGTGCTCATGTCCTCATCC
mus-Il-6	CCAGTTGCCTTCTTGGGACT	GGTCTGTTGGGAGTGGTATCC
mus-Cd206	TTCGGTGGACTGTGGACGAGCA	ATAAGCCACCTGCCACTCCGGT
mus-Arg-1	AACGGGAGGGTAACCATAAGC	TGATGCCCCAGATGGTTTTC
mus-Il-10	CCTCAGTTCCCATTCTATTTATTCACT	TTGAAAGGACACCATAGCAAAGG
mus-Mmp9	AAGGGTACAGCCTGTTCCTGGT	CTGGATGCCGTCTATGTCGTCT
mus-Mmp12	CCAAGCATCCCATCTGCTAT	GGTCAAAGACAGCTGCATCA
mus-Mmp13	TTTATTGTTGCTGCCCATGA	CTCTGGTGTTTTGGGATGCT
mus-Acta2	GTTCAGTGGTGCCTCTGTCA	ACTGGGACGACATGGAAAAG
mus-Col1a1	TAGGCCATTGTGTATGCAGC	ACATGTTCAGCTTTGTGGACC
mus-Col2a1	AGCAGGTCCTTGGAAACCTT	AAGGAGTTTCATCTGGCCCT
mus-Col3	CCTGGAGCCCCTGGACTAATAG	GCCCATTGCACCAGGTTCT
mus-Tgf-b1	CCACCTGCAAGACCATCGAC	CTGGCGAGCCTTAGTTTGGAC
mus-FAP	GGAAGACAAGGTGTATCTGTGG	GTGTTTTCTGCTACTTGAGAATAAT

mus-Pcna	TTTGCACGTATATGCCGAGAC	GGTGAACAGGCTCATTCATCTCT
mus-P50	TAGAATTGCCCCTACCCAGC	AGGAGCAGGACATGGGATTT
mus-Gadd45b	CCTGGCCATAGACGAAGAAG	AGCCTCTGCATGCCTGATAC
mus-Caspase3	GTACAGAGCTGGACTGCGGTATTG	AGTCGGCCTCCACTGGTATCTTC
mus-Gapdh	TACAGCAACAGGGTGGTGGAC	TGGGATAGGGCCTCTCTTGCT
homo-IL-1B	GGACAGGATATGGAGCAACAAG	TCAACACGCAGGACAGGTA
homo-IL-6	AACCTGAACCTTCCAAAGATGG	TCTGGCTTGTTCCTCACTACT
homo-CD206	CTCTGTTCAGCTATTGGACGC	TGGCACTCCCAAACATAATTTGA
homo-IL-10	TAAGCCAGACCTGCCAACCTG	GGTCCTCTGCTAAACGCAAC
homo-GAPDH	GCACCGTCAAGGCTGAGAAC	ATGGTGGTGAAGACGCCAGT

SI References

1. M. Klichinsky *et al.*, "Human chimeric antigen receptor macrophages for cancer immunotherapy" in Nat Biotechnol. (United States, 2020), vol. 38, pp. 947–953.
2. R. Rampado *et al.*, "Lipid Nanoparticles With Fine-Tuned Composition Show Enhanced Colon Targeting as a Platform for mRNA Therapeutics" in Adv Sci (Weinh). (Germany, 2025), vol. 12, pp. e2408744.
3. S. Sabnis *et al.*, A Novel Amino Lipid Series for mRNA Delivery: Improved Endosomal Escape and Sustained Pharmacology and Safety in Non-human Primates. *Mol Ther* **26**, 1509–1519 (2018).

Other supporting materials for this manuscript

Supplementary materials 1

(a modified nucleoside-inclusive mRNA encoding an FAP-targeted CAR sequence)

(Mu) SP -anti-4G5 FAP-scFv-CD28hinge/TM+CD-CD3ζ

ATGGGTGTCCCTACCCAGCTCCTGGGACTGCTCCTGCTGTGGATCACCGACGCCATCT
GCGAC
CAAATTGTTCTCACCCAGTCTCCAGCGCTCATGTCTGCTTCTCCAG
GGGAGAAGGTCACCATGACCTGCACTGCCAGCTCAAGTGTTAGTT
ACATGTACTGGTACCAGCAGAAGCCACGATCCTCCCCCAAACCCT
GGATTTTTCTCACCTCCAACCTGGCTTCTGGAGTCCCTGCTCGCTT
CAGTGGCCGTGGGTCTGGGACCTCTTTCTCTCTCACAATCAGCAG
CATGGAGGCTGAAGATGCTGCCACTTATTACTGCCAGCAGTGGAG
TGGTTACCCACCCATCACATTCGGCTCGGGGACAAAGTTGGAAAT
AAAAGGTGGAGGTGGCAGCGGAGGAGGTGGGTCCGGCGGTGGA
GGAAGCCAGGTCCAACTGCAGCAGCCTGGGGCTGAACTGGTAAA
GCCTGGGGCTTCAGTGAAGTTGTCCTGCAAGGCGTCTGGCTACAC
CATCACCAGCTACTCTCTGCACTGGGTGAAGCAGAGGCCTGGACA
AGGCCTTGAGTGGATTGGAGAGATTAATCCTGCCAATGGTGATCA

TAACTTCAGTGAGAAGTTCGAGATCAAGGCCACACTGACTGTAGA
CAGCTCCTCCAACACAGCATTCATGCAACTCAGCAGGCTGACATC
TGAGGACTCTGCGGTCTATTACTGTACAAGATTGGACGATAGTAG
GTTCCACTGGTACTTCGATGTCTGGGGCGCAGGGACCACGGTCAC
CGTCTCCTCA
ATCGAGTTCATGTACCCCCCTCCCTACCTGGACAACGAGAGAAGCAACGGCACCATCA
TCCACATCAAAGAAAAGCACCTGTGCCACACCCAGAGCAGCCCCAAGCTGTTCTGGG
CCCTGGTGGTGGTGGCCGGCGTGCTGTTCTGTTACGGCCTGCTGGTCACAGTGGCCCT
GTGCGTGATCTGGACCAACAGCAGAAGAAACAGAGGCGGCCAGAGCGACTACATGAA
CATGACCCCCAGAAGGCCAGGCCTGACCAGAAAGCCCTACCAGCCCTACGCCCCTGC
CAGAGACTTCGCCGCCTACAGACCC
AGAGCCAAGTTCAGCAGATCCGCCGAGACAGCCGCCAACCTGCAGGATCCCAACCAG
CTGTTCAACGAGCTGAACCTGGGCAGACGGGAGGAATTCGACGTGCTGGAAAAGAAG
AGAGCCAGGGACCCCGAGATGGGCGGCAAGCAGCAGAGAAGAAGAAACCCTCAGGA
AGGCGTCTACAACGCCCTGCAGAAAGACAAGATGGCCGAGGCCTACAGCGAGATCGG
CACCAAGGGCGAGAGAAGAAGGGGCAAGGGCCACGATGGCCTGTTCCAGGGCCTGT
CCACCGCCACCAAGGACACCTTCGACGCCCTGCACATGCAGACCCTGGCCCCCAGATA
A
